# *MEG3*-derived miR-493-5p overcomes the oncogenic feature of *IGF2*-miR-483 loss of imprinting in hepatic cancer cells

**DOI:** 10.1038/s41419-019-1788-6

**Published:** 2019-07-18

**Authors:** Luc Gailhouste, Lee Chuen Liew, Ken Yasukawa, Izuho Hatada, Yasuhito Tanaka, Takashi Kato, Hitoshi Nakagama, Takahiro Ochiya

**Affiliations:** 1 Division of Molecular and Cellular Medicine, National Cancer Center Research Institute, Tokyo, Japan; 2 Graduate School of Medicine, The University of Tokyo, Tokyo, Japan; 3 Department of Integrative Bioscience and Biomedical Engineering, Graduate School of Advanced Science and Engineering, Waseda University, Tokyo, Japan; 4 Laboratory of Genome Science, Biosignal Genome Resource Center, Institute for Molecular and Cellular Regulation, Gunma University, Maebashi, Japan; 5 Department of Virology and Liver Unit, Nagoya City University Graduate School of Medical Sciences, Nagoya, Japan; 6 National Cancer Center, Tokyo, Japan; 7 Present Address: Liver Cancer Prevention Research Unit, RIKEN Center for Integrative Medical Sciences, 2-1 Hirosawa, Wako, Saitama, 351-0198 Japan; 8 Present Address: Department of Molecular and Cellular Medicine, Institute of Medical Science, Tokyo Medical University, 6-7-1 Shinjuku-ku, Tokyo, 160-0023 Japan

**Keywords:** Cancer therapy, Liver cancer, DNA methylation, Imprinting, miRNAs

## Abstract

Numerous studies have described the critical role played by microRNAs (miRNAs) in cancer progression and the potential of these small non-coding RNAs for diagnostic or therapeutic applications. However, the mechanisms responsible for the altered expression of miRNAs in malignant cells remain poorly understood. Herein, via epigenetic unmasking, we identified a group of miRNAs located in the imprinted delta like non-canonical Notch ligand 1 (*DLK1*)-maternally expressed 3 (*MEG3*) locus that were repressed in hepatic tumor cells. Notably, miR-493-5p epigenetic silencing was correlated with hypermethylation of the *MEG3* differentially regulated region (DMR) in liver cancer cell lines and tumor tissues from patients. Experimental rescue of miR-493-5p promoted an anti-cancer response by hindering hepatocellular carcinoma (HCC) cell growth in vitro and tumor progression in vivo. We found that miR-493-5p mediated part of its tumor-suppressor activity by abrogating overexpression of insulin-like growth factor 2 (*IGF2*) and the *IGF2*-derived intronic oncomir miR-483-3p in HCC cells characterized by *IGF2* loss of imprinting (LOI). In summary, this study describes an unknown miRNA-dependent regulatory mechanism between two distinct imprinted loci and a possible therapeutic window for liver cancer patients exhibiting *IGF2*-miR-483 LOI and amplification.

## Introduction

Advances in the understanding of epigenetic regulatory mechanisms and their implications in tumor progression have greatly contributed to the development of epigenome-based cancer research^1^. At the molecular level, CpG methylation and histone modifications are considered the canonical epigenetic processes, which are fundamental for regulating and orchestrating various cellular functions^2^. Tumorigenesis is generally associated with a global decrease in DNA methylation, especially in gene bodies and intergenic regions, leading to genomic instability or loss of imprinting (LOI)^3^. However, cancer cells also exhibit selective hypermethylation restricted to gene promoters and CpG islands, which can result in silencing of tumor-suppressors^4^. Overall, the reversible nature of epigenetic modifications makes abnormal DNA methylation features potentially more ‘druggable’ than conventional genome alterations resulting from mutations.

MicroRNAs (miRNAs) constitute a group of conserved small non-coding RNAs that regulate gene expression by complementary base pairing with the 3′-untranslated region (3′-UTRs) of messenger RNAs (mRNAs)^5^. Abnormal miRNA expression has been extensively reported and is considered a hallmark of human cancer^6,7^. However, the regulatory networks responsible for inactivation of tumor-suppressor miRNAs or reactivation of critical oncomirs remain poorly understood and require further investigation. For this purpose, primary liver cancer appeared to be a relevant model for the study of epigenetically altered antitumor miRNAs. Indeed, the accumulation of major epigenetic aberrations, such as DNA hypermethylation, is typically observed during precancerous stages in patients suffering from chronic liver disease^8^ and more dramatically in hepatic tumors^9,10^.

Taking advantage of an epigenetic unmasking approach used to reverse genomic hypermethylation marks, we highlighted a set of six miRNAs epigenetically silenced in hepatocellular carcinoma (HCC) cells and located in the imprinted delta like non-canonical Notch ligand 1 (*DLK1*)-maternally expressed 3 (*MEG3*) locus, in which miR-493-5p repression was correlated with *MEG3*-differentially regulated region (DMR) methylation. Notably, experimental rescue of miR-493-5p promoted an anti-cancer response by negatively regulating the expression of insulin-like growth factor 2 (*IGF2*)-derived oncomir miR-483-3p in liver cancer cell lines exhibiting *IGF2* LOI.

## Results

### Epigenetic unmasking revealed a subset of miRNAs silenced in the imprinted *DLK1*-*MEG3* locus

**Fig. 1:**
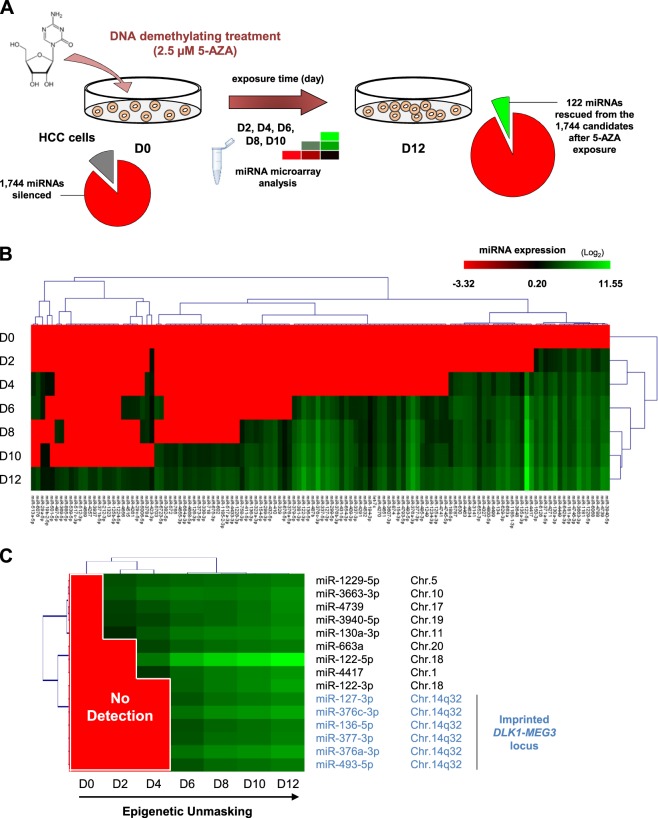
Epigenetic unmasking of *MEG3*-derived miRNAs. **a** Experimental design of miRNA epigenetic unmasking. The cartoon illustrates the strategy used to identify miRNAs epigenetically silenced in liver cancer cells. **b** Expression profiles of the 122 miRNAs rescued in HepG2 cells in response to 5-AZA demethylating treatment (2.5 µM). Cells were collected every 2 days from days 0 (D0) to 12 (D12), and RNAs were extracted for microarray analysis. The scale bar encodes the logarithm of relative miRNA expression levels. miRNAs that were expressed before epigenetic unmasking were excluded from the analysis. (GSE123314) **c** List of the miRNAs exhibiting the most significant re-expression after epigenetic unmasking (top 15). miR-127-3p, miR-136-5p, miR-376-a-3p, miR-376c-3p, miR-377-3p, and miR-493-5p were found to be located on chromosome 14 and associated with the imprinted *DLK1*-*MEG3* locus

To identify tumor-suppressor miRNAs epigenetically silenced in liver cancer, the human cell line HepG2 was treated with the demethylating agent 5-azacytidine (5-AZA) for 12 days (epigenetic unmasking). First, microarray expression profiles revealed that 1744 miRNAs were silenced in the control HepG2 cells (Fig. 1a). Among these 1744 miRNAs, 122 were found to be significantly re-expressed by more than 2-fold after epigenetic treatment (Fig. 1b). We observed that the canonical hepatic tumor-suppressor miRNA, miR-122, which is known to be silenced in HCC cell lines and hepatic cancer tissues^11^, was strongly induced after epigenetic unmasking. Of the 15 rescued miRNAs that exhibited the highest expression levels after 12 days of treatment, 6 were derived from the imprinted *DLK1*-*MEG3* locus located on chromosome 14q32 (Fig. 1c).

**Fig. 2:**
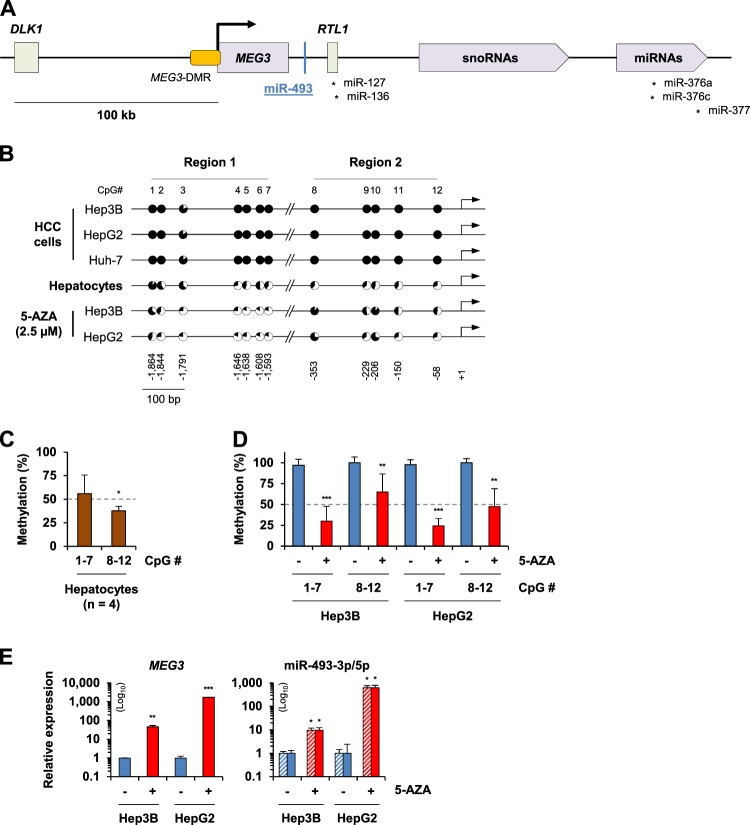
Methylation and expression profiles of the *MEG3*-miR-493-5p locus after epigenetic unmasking. **a** Schematic representation of the imprinted *DLK1*-*MEG3* locus on human chromosome 14. This genomic region consists of protein-coding genes (including *DLK1* and retrotransposon Gag like 1 (*RTL1*)), lncRNAs (including *MEG3*), small nucleolar RNAs (snoRNAs), and miRNAs. While *DLK1* and *RTL1* are paternally expressed, *MEG3* shows maternal expression. *MEG3*-DMR covers a genomic segment upstream of the *MEG3* TSS and extending to its first exon. The black arrow indicates the *MEG3* TSS. The position of the six miRNAs highlighted after epigenetic unmasking is depicted. **b** Comparison of the *MEG3*-DMR methylation level between hepatic tumor cells and human hepatocytes. COBRA was performed to evaluate methylation of the CpG sites located upstream of the *MEG3* TSS. Twelve CpG sites were analyzed in two distinct CpG-rich regions of *MEG3*-DMR, ranging from −1864 to −1593 bp for Region 1 (CpG #1 to #7) and −353 to −58 bp for Region 2 (CpG #8 to #12). The circles represent in black the methylation ratio calculated for each CpG analyzed in HCC cells (*n* = 3 per CpG). The average values of CpG methylation levels measured in human hepatocytes from four different donors were used as a reference. **c** Quantification of *MEG3*-DMR methylation status evaluated by COBRA in human hepatocytes. The methylation ratios (%) represent the average values calculated for CpG #1 to #7 and CpG #8 to #12, which delimited Region 1 and Region 2, respectively. **d** Quantification of *MEG3*-DMR methylation status after 5-AZA treatment, evaluated by COBRA in Hep3B and HepG2 cells. Genomic DNA was extracted after demethylating treatment with 2.5 µM 5-AZA for 10 days. **e** Expression levels of *MEG3*, miR-493-3p, and miR-493-5p after epigenetic unmasking. The relative mRNA and mature miRNA expression levels were determined by RT-qPCR. Non-treated HCC cells were used as controls. The histograms shown in the figure represent the mean ± SD. Significant differences in gene expression and methylation levels were achieved at *p < 0.05, **p < 0.01, and ***p < 0.001 using a *t*-test and Mann–Whitney *U* test, respectively

The imprinted *DLK1*-*MEG3* cluster contains paternally expressed protein-coding genes, including *DLK1*, and various maternally expressed non-coding genes, such as the long non-coding RNA (lncRNA) *MEG3* and numerous miRNAs^12^ (Fig. 2a). These maternally expressed non-coding RNAs are all processed from a single primary transcript that initiates from the *MEG3* promoter^13^. Loss of *MEG3* expression because of DMR hypermethylation has been reported in various diseases and malignancies^14–16^, including primary liver cancer^17^. We identified miR-493-5p as one of the most significantly re-expressed *MEG3*-derived miRNAs after epigenetic unmasking. In line with the microarray findings, reverse transcription-quantitative polymerase chain reaction (RT-qPCR) analysis confirmed the re-expression of miR-493-5p in response to 5-AZA demethylating treatment, whereas this miRNA was not detectable in the control cells (Supplementary Fig. 1A). Further investigations were considered in order to clarify miR-493-5p regulatory networks in the liver neoplastic model.

### miR-493-5p silencing is correlated with *MEG3*-DMR hypermethylation in hepatic cancer cells

Using combined bisulfite restriction analysis (COBRA), we evaluated the methylation level of CpG sites located upstream of the *MEG3* transcription start site (TSS) in three HCC cell lines. First, the COBRA data evidenced a dramatic hypermethylation of all of the CpGs analyzed in the three cell lines (Fig. 2b). The average methylation ratio measured in Hep3B, HepG2, and Huh-7 cells was 98.6 ± 0.3%. Control DNA extracted from hepatocytes of four different donors also exhibited a marked methylation ratio (Fig. 2c). In line with these observations, the difficulty in quantifying *MEG3* and miR-493-5p expression by RT-qPCR (data not shown) supported the hypothesis that an epigenetic mechanism was most likely responsible for *MEG3*-miR-493 silencing in normal and transformed hepatic cells.

To confirm that miR-493-5p is epigenetically silenced in liver cancer cells, similar CpG sites were analyzed via COBRA after exposure to the demethylating agent 5-AZA. Epigenetic treatment promoted a major demethylation of *MEG3*-DMR in Hep3B and HepG2 cells, with a decrease of 44.7 ± 25.7% and 33.9 ± 18.8%, respectively (Fig. 2d). More precisely, we observed that Region 1 (distant from TSS) exhibited lower methylation levels than Region 2 (adjacent to TSS), with 30.2 ± 17.5% (Region 1) versus 65.0 ± 21.6% (Region 2) and 24.3 ± 8.8% (Region 1) versus 47.4 ± 21.6% (Region 2), in the treated Hep3B and HepG2 cells, respectively. Exposure of human hepatocytes to the epigenetic agent did not modify CpG methylation status (Supplementary Fig. 2B). Together, these results suggest that methylation of the distal CpGs of *MEG3*-DMR (Region 1) could be critical for maintenance of miR-493 epigenetic silencing rather than the CpG sites directly adjacent to *MEG3* TSS.

Next, we analyzed the expression of *MEG3* and mature miR-493-3p/5p in liver cancer cells after demethylating treatment. A marked re-expression of *MEG3* and miR-493-3p/5p was evidenced in Hep3B, HepG2 (Fig. 2e), and Huh-7 cells (Supplementary Fig. 2C). To strengthen the results obtained after epigenetic unmasking, *MEG3* and miR-493 methylation and expression profiles were analyzed after knockdown of DNA methyltransferase 1 (*DNMT1*), which is one of the major enzymes that control DNA methylation^2^. *DNMT1* expression is also known to be dramatically increased in hepatic cancer cells^18^, leading to tumor-suppressor gene hypermethylation^19^. The effect of *DNMT1* knockdown was consistent with the reversion of *MEG3* and miR-493-3/5p epigenetic silencing observed after 5-AZA treatment (Supplementary Fig. 3).

### Epigenetic silencing of miR-493-5p is a mark of advanced liver cancer

**Fig. 3:**
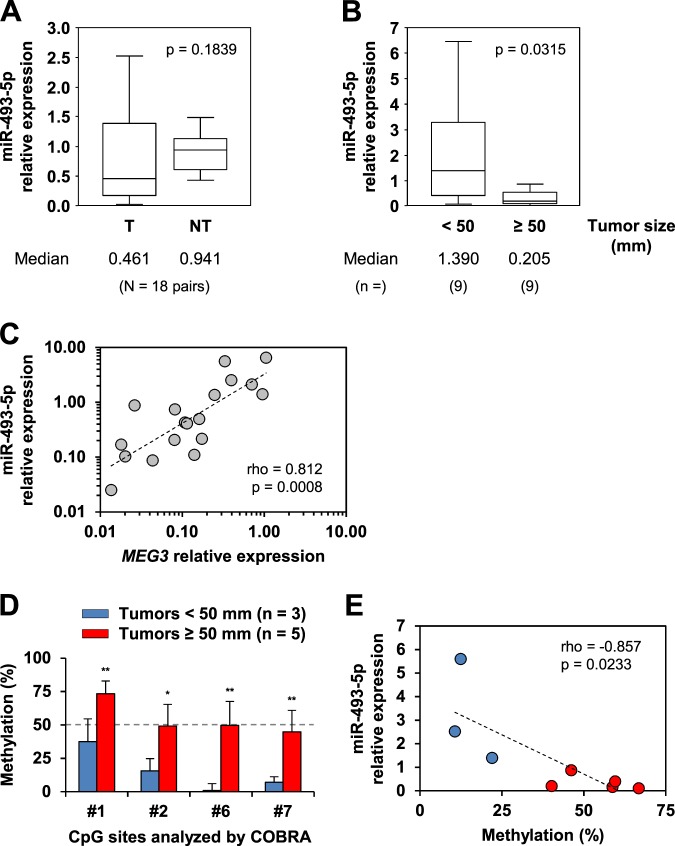
Assessment of miR-493-5p expression and *MEG3*-DMR methylation in clinical samples from HCC patients. **a** Expression levels of miR-493-5p in clinical samples. The boxplots illustrate differential gene expression in 18 primary HCC tumors (T) compared with their corresponding non-tumor tissues (NT). Mann–Whitney *U* test was used to calculate the *p*-value. **b** Comparison of miR-493-5p expression between moderate and advanced liver cancer. Tumors with a size superior or equal to 5 cm were considered advanced HCC tumors. Mann–Whitney *U* test indicated a significant decrease in the miR-493-5p level in the group of patients with advanced tumors (*p* < 0.05). **c** Scatter plots of Spearman’s correlation coefficient analysis between miR-493-5p and *MEG3* relative expression, measured by RT-qPCR in HCC tumor tissues (*n* = 18). **d** Comparison of *MEG3*-DMR methylation levels between moderate and advanced liver cancer. Methylation levels of four CpG sites (Region 1) were randomly evaluated via COBRA in advanced (*n* = 5) and moderate tumors (*n* = 3). Histograms show the mean ± SD. Significance is indicated as *p < 0.05 and **p < 0.01 (*t*-test). **e** Correlation analysis between miR-493-5p expression and *MEG3*-DMR methylation levels in HCC tumors (Spearman’s rank coefficient). The red and blue plots show advanced tumors and moderate tumors, respectively

To assess whether miR-493-5p expression could be quantified in clinical samples from liver cancer patients, RT-qPCR was performed. The data revealed that miR-493-5p expression levels were accurately measurable and globally reduced by ~2-fold in HCC tumors compared with their adjacent surrounding non-neoplastic tissues (median, 0.461 and 0.941, respectively; *p* = 0.1839, Mann–Whitney *U* test; Fig. 3a). Notably, miR-493-3p and *MEG3* expression was inhibited in a more significant manner in HCC tumors: 0.291 versus 0.858 (*p* = 0.0104) and 0.127 versus 0.887 (*p* < 0.0001), respectively (Supplementary Fig. 4).

We tested whether miR-493-5p inhibition was correlated with tumor progression. For this purpose, HCC biopsies were separated into two groups based on their clinical features: tumor size inferior to 5 cm (*n* = 9) and tumor size superior or equal to 5 cm (*n* = 9) (Supplementary Table 1). Remarkably, miR-493-5p expression appeared to be dramatically decreased in the group of patients with advanced HCC tumors (median, 0.205 versus 1.390; *p* = 0.0315, Mann–Whitney *U* test; Fig. 3b). In addition, miR-493-5p and *MEG3* expression levels were clearly correlated in the 18 HCC tumors, with a Spearman’s coefficient of rank correlation *ρ* value of 0.812 (*p* = 0.0008; Fig. 3c). We randomly analyzed the methylation ratios of four CpG sites located in *MEG3*-DMR (Region 1) with the aim of establishing a possible correlation between miR-493-5p silencing and *MEG3* hypermethylation in clinical samples. The results revealed that patients with advanced tumors exhibited higher methylation levels than those with moderate HCC (Fig. 3d). Furthermore, *MEG3*-DMR methylation was significantly correlated with a decrease in miR-493-5p expression (*ρ* = −0.857; *p* = 0.0233; Fig. 3e).

### miR-493-5p rescue promotes a tumor-suppressor effect in hepatic cancer cells

**Fig. 4:**
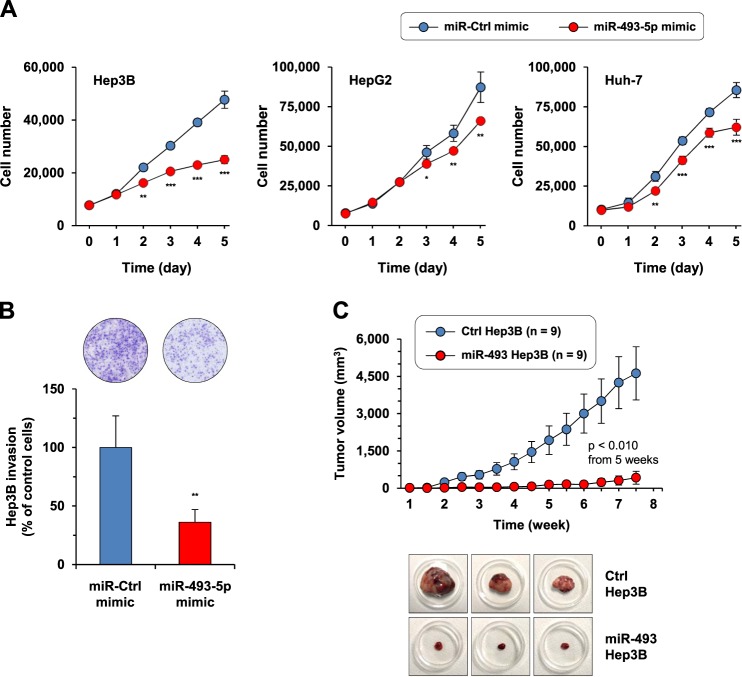
Effect of miR-493-5p rescue on hepatic cancer cell malignancy. **a** Tumor cell growth evaluated after miR-493-5p rescue. The number of cells was estimated after miR-493-5p overexpression using cell viability assays. Cells transfected with control mimics (miR-Ctrl mimic) were used as a reference. The data represent the mean ± SD. **b** Invasive abilities of Hep3B cells after miR-493-5p re-expression. Cells that migrated through the Matrigel-coated membrane were counted after 72 h. The histograms show the mean ± SD. **c** HCC tumor growth in response to miR-493-5p rescue. The data show the mean ± SEM calculated for tumors in the control (*n* = 9) and miR-493 group (*n* = 9). The pictures show representative tumor sizes at the end of the experiment: 4619.6 ± 1072.1 mm^3^ and 422.7 ± 86.1 mm^3^ for the control (Ctrl Hep3B) and miR-493 group (miR-493 Hep3B), respectively. Significant differences in cell migration and tumor growth (in vitro and in vivo) were evaluated with a *t*-test: *p < 0.05, **p < 0.01, and ***p < 0.001

Next, we evaluated the putative tumor-suppressor activity of miR-493-5p. For this purpose, miRNA mimics were used to rescue miR-493-5p and enabled significant re-expression of the miRNA in liver cancer cell lines (Supplementary Fig. 1B). First, viability assays showed that HCC cell growth was consistently inhibited after miR-493-5p overexpression compared with the control cells that did not express the miRNA (Fig. 4a). Hep3B cells exhibited the most marked inhibition, which was equivalent to a growth reduction of 47.8 ± 3.2% relative to the control cells (*p* < 0.001, *t*-test at day 5). HepG2 and HuH-7 cell growth was decreased to a lesser extent by ~25%. We also analyzed the invasive abilities of Hep3B and Huh-7 cells via transwell migration assays and found that miR-493-5p rescue remarkably altered Hep3B cell invasion (inhibition of 63.9 ± 19.2%; *p* = 0.0096, *t*-test; Fig. 4b). This inhibition was more limited in Huh-7 cells (34.2 ± 12.8%; *p* = 0.0691, *t*-test; Supplementary Fig. 5). Last, cell viability measurement revealed that miR-493-5p mimics did not improved drug-response given that Hep3B cells remained resistant to sorafenib treatment after miR-493-5p rescue (Supplementary Fig. 6).

To strengthen our in vitro data, the tumor-suppressor effect of miR-493-5p was tested using an in vivo tumor growth model. Hep3B cells with stable re-expression of miR-493-5p were generated (Supplementary Fig. 7). Next, control and miR-493 cells were subcutaneously implanted into nude mice, and tumor growth was monitored. At the end of the experiment, tumors resected from mice that received cells overexpressing mir-493-5p appeared markedly smaller than those from control group mice: 422.7 ± 86.1 mm^3^ and 4619.6 ± 1072.1 mm^3^, respectively (Fig. 4c). Xenograft monitoring showed a very low growth rate for the miR-493 tumors (*p* < 0.01 from week 5, *t*-test), and the nodules remained nearly undetectable for up to 4 weeks after cell implantation.

### *IGF2* expression is regulated by miR-493-5p through 3′-UTR targeting

**Fig. 5:**
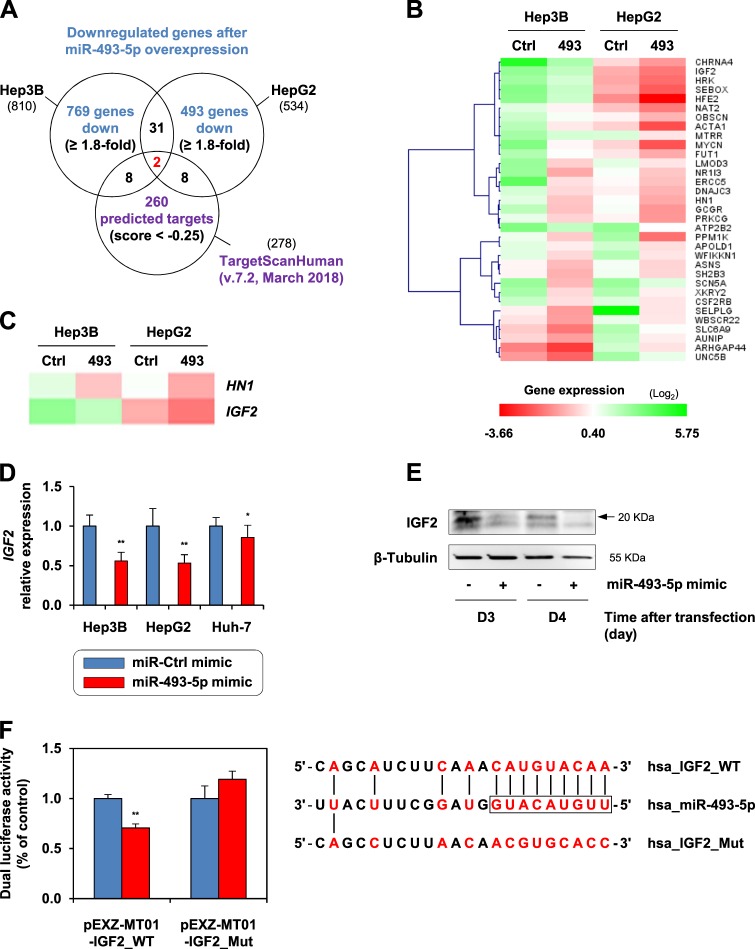
Identification of miR-493-5p targets. **a** Venn diagram summarizing the experimental approach used to identify potential miR-493-5p targets in HCC cells. **b** Expression pattern of the 33 genes downregulated after re-expression of miR-493-5p. The scale bar encodes the logarithm of relative mRNA levels measured in Hep3B and HepG2 cells. (GSE123313) **c** Putative targets of miR-493-5p. *HN1* and *IGF2* were selected based on the microarray data and in silico analysis using the online miRNA target prediction tool TargetScanHuman. **d** Relative expression of *IGF2* following miR-493-5p rescue in HCC cells. Gene expression levels were measured 72 h after transfection with control and miR-493-5p mimics. **e** IGF2 protein levels determined by immunoblotting after miR-493-5p overexpression in Hep3B cells. Whole-cell lysates were collected 72 and 96 h after transfection. IGF2 was detected using a primary antibody against the human IGF2 precursor (20 KDa). β-Tubulin was used as the loading control. **f** IGF2 3′-UTR assay. A dual luciferase reporter assay was performed using Hep3B cells after cotransfection with miR-493-5p mimics and the firefly-Renilla luciferase construct containing the human IGF2 3′-UTR. A vector containing a mutated sequence of IGF2 3′-UTR was used as the negative control. The luciferase activities were measured 24 h after transfection, and the ratio of firefly/Renilla luciferase activities was determined. The depicted sequences indicate the complementarity between miR-493-5p and IGF2 3′-UTR. All histograms shown in the figure represent the mean ± SD. Significance: *p < 0.05 and **p < 0.01 (*t*-test)

To identify the targets by which miR-493-5p mediates its tumor-suppressor activity, global gene expression was analyzed in HepB3 and HepG2 cells after miR-493-5p rescue. From the microarray data, we extracted 810 and 534 genes in Hep3B and HepG2 cells, respectively, given their significant downregulation in response to miR-493-5p overexpression (Fig. 5a). Thirty three genes were consistently inhibited in both cell lines (Fig. 5b). Next, we used the online miRNA target prediction tool TargetScanHuman to refine our list of putative targets. Among 278 predicted targets, 2 genes appeared downregulated in our microarray data (Fig. 5c): hematological and neurological expressed 1 (*HN1*) and *IGF2*. We also performed Gene Set Enrichment Analysis (GSEA) and observed relevant enrichment of three sets of genes in HCC cells overexpressing miR-493-5p, which was consistent with the antitumor effect of the microRNA (Supplementary Fig. 8).

Based on a relevant literature that demonstrated an oncogenic role of IGF2 in liver cancer^10,20^, we further investigated the interaction between *IGF2* and miR-493-5p. miR-493-5p rescue clearly affected *IGF2* expression, which was decreased by 44.0 ± 8.5%, 46.6 ± 9.1%, and 14.3 ± 2.6% in Hep3B, HepG2, and Huh-7 cells, respectively (Fig. 5d). Immunoblotting confirmed that miR-493-5p overexpression also decreased IGF2 at the protein level in Hep3B cells (Fig. 5e). Furthermore, an inverse correlation was established between miR-493-5p and *IGF2* expression levels using in vivo data from the Hep3B tumors (*ρ* = −0.742; *p* = 0.0022; Supplementary Fig. 7E). Last, we found that *IGF2* contained a sequence in its 3′-UTR that was partially complementary to miR-493-5p (Fig. 5f). Simultaneous transfection with miR-493-5p mimics and a reporter vector that contained the human *IGF2* 3′-UTR was performed using Hep3B cells. The 3′-UTR assay showed that forced expression of miR-493-5p decreased luciferase activity by 29.3 ± 1.7% from the value obtained with the control mimics (*p* = 0.006; *t*-test; Fig. 5f). Conversely, we did not observe inhibition of reporter activity in the cells transfected with a vector that contained a mutated sequence of the *IGF2* 3′-UTR.

### miR-483-3p overexpression is correlated with *IGF2* LOI in liver cancer cells

**Fig. 6:**
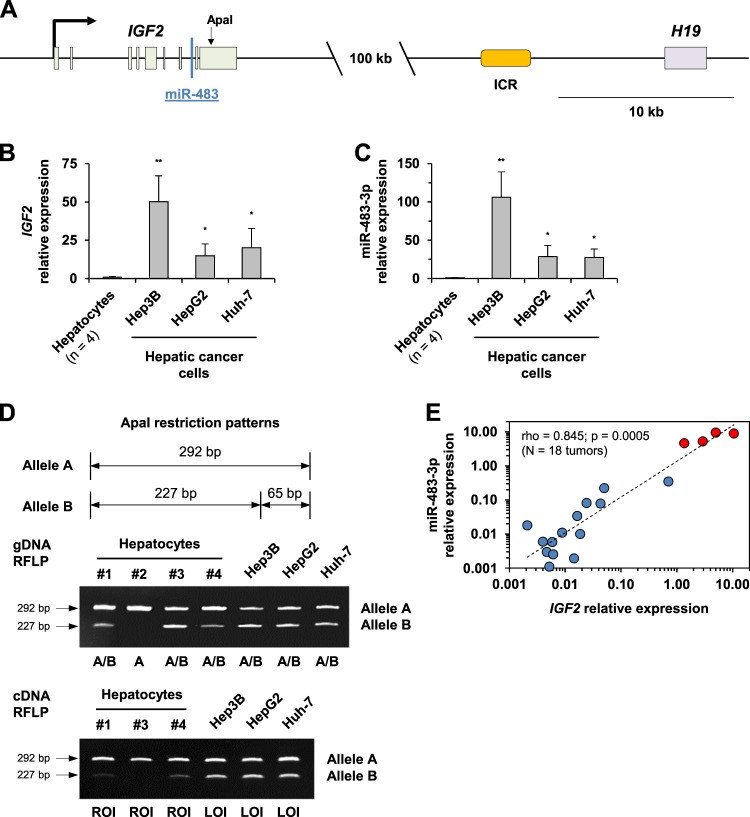
*IGF2* and *IGF2*-derived intronic miR-483-3p expression status. **a** Schematic representation of the imprinted *H19*-*IGF2* locus. *IGF2* and *H19* lncRNA are coordinately regulated by an intergenic control region (ICR) and a common enhancer region downstream of *H19*. In humans, *IGF2* is maternally imprinted, while *H19* exhibits paternal imprinting. *IGF2* contains nine exons and five transcription variants. miR-483 is an encoded miRNA located within the second intron of *IGF2*. The black arrow indicates the *IGF2* TSS for transcript variant 2. All five transcript variants contain miR-483. **b**, **c** Expression levels of *IGF2* (**b**) and *IGF2*-derived intronic miR-483-3p (**c**) in hepatic tumor cells and human hepatocytes. The average values of *IGF2* and mature miR-483-3p expression levels measured in human hepatocytes from four different donors were used as a reference. The histograms represent the mean ± SD. Statistical differences relative to control hepatocytes were reached at *p < 0.05 and **p < 0.01 using a *t*-test. **d** Allelic expression of *IGF2* in Hep3B, HepG2, and Huh-7 cells and normal hepatocytes based on ApaI digestion of a single-nucleotide polymorphism on *IGF2* exon 9. For *IGF2* genotyping (gDNA RFLP), ApaI-digested PCR products were labeled A/B for heterozygous cases, which were considered as informative and eligible for imprinting analysis. One lot of hepatocytes (#2) was homozygous for *IGF2* and excluded. For imprinting status analysis (cDNA RFLP), ApaI-digested RT-PCR products indicated biallelic expression (LOI) when ratios of the most abundant allele to the less abundant allele were lower than 2. ROI, retention of imprinting. **e** Scatter plots of Spearman’s correlation coefficient analysis between miR-483-3p and *IGF2* expression levels, measured by RT-qPCR in the 18 tumor samples from HCC patients. The red and blue plots show tumors with high (*n* = 4) and low (*n* = 14) expression levels of *IGF2* and miR-483-3p, respectively

*IGF2* belongs to a cluster of genes located on chromosome 11p15.5 (Fig. 6a). Similar to the *MEG3*-miR-493 cluster, the *H19*-*IGF2* locus is controlled by genomic imprinting, in which *IGF2* is paternally expressed, while its maternal allele is repressed^21^. *IGF2* LOI has been extensively described in various types of tumors^22,23^, including liver cancer^24–26^. Interestingly, the oncogenic activity of mature miR-483-3p that originates from *IGF2*-derived intronic primary miR-483 (pri-miR-483) has also been well reported^27^. We first evidenced a concomitant and marked overexpression of *IGF2* (Fig. 6b) and miR-483-3p (Fig. 6c) in liver cancer cell lines. Consistently, genotyping and imprinting status analysis of the *IGF2* gene using restriction fragment length polymorphism (RFLP) confirmed *IGF2* retention of imprinting (ROI) in hepatocytes, whereas Hep3B, HepG2, and Huh-7 cells exhibited *IGF2* LOI and biallelic expression (Fig. 6d). We found that demethylating treatment was able to reverse *IGF2*-miR-483 locus overexpression in tumor cells, evidenced by the decreased expression of *IGF2* and miR-483-3p/5p in response to 5-AZA exposure (Supplementary Fig. 9). In accordance with previous studies that reported *IGF2* LOI incidence and *IGF2*-miR-483 amplification in ~15% of primary liver tumors^20,24,27^, we observed overexpression of *IGF2* and miR-483-3p in 4 out of 18 tumors obtained from HCC patients (Supplementary Fig. 4E). A significant correlation between *IGF2* and miR-483-3p expression was established in these samples (*ρ* = 0.845; *p* < 0.001; Fig. 6e). Using a dataset from Gene Expression Omnibus (GEO) (GSE74618), we also verified the inverse correlation between miR-493-5p and miR-483-3p in HCC clinical samples exhibiting high miR-493-5p expression levels (Supplementary Fig. 10).

### miR-493-5p rescue suppresses tumor cell growth by inhibiting the *IGF2*-derived intronic miR-483-3p

**Fig. 7:**
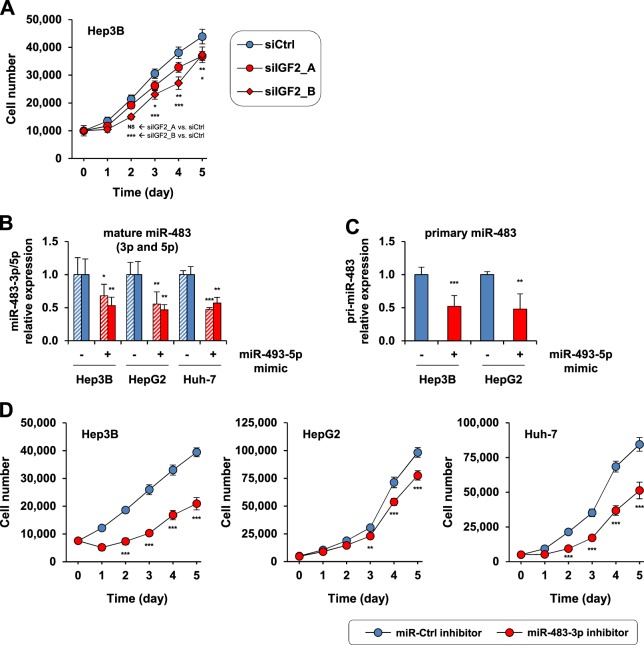
Tumor cell growth regulatory mechanism mediated by miR-493-5p through *IGF2*-miR-483-3p inhibition. **a** Tumor cell growth evaluated after *IGF2* knockdown. Two distinct siRNAs were used to target IGF2 (siIGF2_A and siIGF2_B) in Hep3B cells, and scrambled siRNAs were used as negative controls. The number of cells was estimated at the indicated times using a cell viability assay. HepG2 and Huh-7 cells were considered resistant to *IGF2* silencing given the absence of cell growth inhibition after transfection (Supplementary Fig. 11). **b** Mature miR-483 (3p and 5p) and **c** pri-miR-483 expression levels after experimental re-expression of miR-493-5p in HCC cells. Total RNA was extracted 72 h after cell transfection for RT-qPCR analysis. **d** Tumor cell growth assessment after miR-483-3p knockdown. Hep3B, HepG2, and Huh-7 cell growth was determined after transfection with miR-483-3p inhibitors (see Supplementary Fig. 1C for miR-483-3p inhibition control). Cells transfected with control inhibitors were used as a reference. All experimental data shown in the figure represent the mean ± SD. Statistical differences in miRNA expression measurement and cell growth assays relative to control siRNAs or control miRNA mimics and inhibitors were evaluated with a *t*-test: *p < 0.05, **p < 0.01, and ***p < 0.001. NS, not significant

**Fig. 8:**
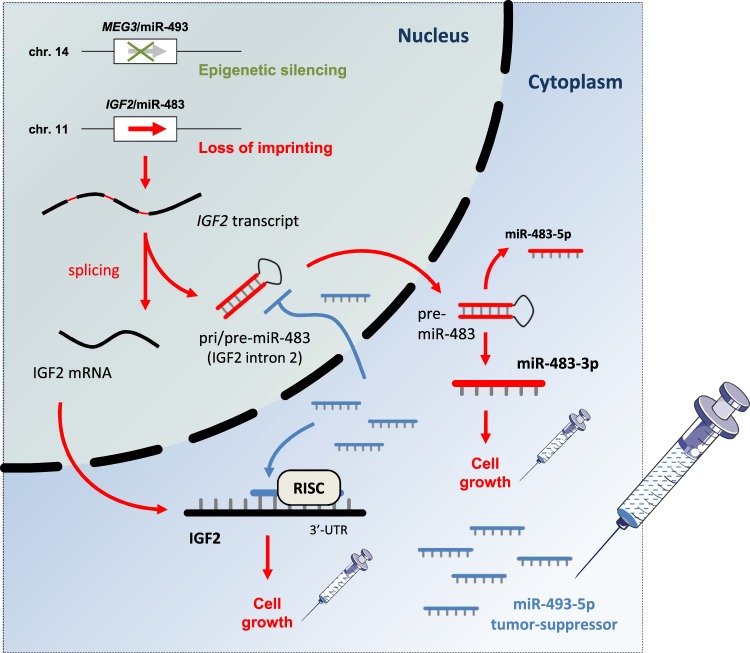
*MEG3*-derived miR-493-5p overcomes the oncogenic feature of *IGF2*-miR-483 loss of imprinting in hepatic cancer cells. Proposed regulatory network in which the rescue of epigenetically silenced miR-493-5p inhibits growth properties of liver cancer cells exhibiting *IGF2*-miR-483 LOI

To clarify the impact of the miR-493-5p-*IGF2* axis in liver cancer cells, we tested whether *IGF2* experimental silencing could mimic the tumor-suppressor activity mediated by miR-493-5p. While *IGF2* knockdown inhibited the invasive properties of Hep3B cells (Supplementary Fig. 11B), the impact of *IGF2* repression on Hep3B cell growth was more limited (Fig. 7a). Based on these results, we postulated that miR-493-5p could mediate a more important part of its growth inhibitory activity through inhibition of *IGF2*-derived miR-483-3p. First, mature miR-483-3p levels were analyzed after miR-493-5p overexpression to test this hypothesis. An inverse correlation between miR-483-3p and miR-493-5p was demonstrated given the marked decrease in miR-483-3p expression levels after miR-493-5p re-expression in HCC cell lines (Fig. 7b). Given that miR-493-5p rescue modulated the expression of both mature miR-483-3p and miR-483-5p, we tested whether pri-miR-483 was also affected. As expected, miR-493-5p overexpression decreased pri-miR-483 expression level (Fig. 7c), suggesting a potential regulation mechanism that occurs before pri-miR-483 processing. Finally, the effect of miR-483-3p inhibition on tumor cell growth was evaluated. Our data indicated that miR-483-3p experimental knockdown significantly decreased cell growth by 47.0 ± 5.7%, 21.1 ± 4.3%, and 39.3 ± 7.1% in Hep3B, HepG2, and Huh-7 cells, respectively (*p* < 0.001, *t*-test at day 5; Fig. 7d). Combined, these results argue for the existence of a regulatory network between two imprinted genomic loci, in which miR-493-5p rescue can overcome the oncogenic activity of *IGF2*-derived intronic miR-483 in cancer cells exhibiting *IGF2*-miR-483 LOI (Fig. 8).

## Discussion

In this study, we highlighted an interconnection between two epigenetically controlled miRNAs located in two distinct imprinted loci: (1) *MEG3*-derived miR-493-5p, which was silenced through *MEG3*-DMR hypermethylation in liver cancer cells and tumors from patients, and (2) *IGF2*-derived intronic miR-483-3p, which was overexpressed in hepatic cell lines exhibiting *IGF2* LOI.

Various studies have reported the tumor-suppressor function of *MEG3* and its deregulated expression in cancer cells^15,28^. Anwar et al.^29^ analyzed *MEG3* expression in a series of 34 human HCC specimens and observed that 20 HCC samples displayed downregulation of *MEG3*. More interestingly, a significant correlation between *MEG3*-DMR methylation and reduction of *MEG3* expression was established in these samples, which was consistent with our observations. More recently, Zheng et al.^30^ demonstrated that MEG3 inhibited the malignant phenotype of liver cancer cells.

Thus far, the most well-studied imprinted region in the mammalian genome is likely the *H19*-*IGF2* locus. Our data showed that upon miR-493-5p rescue, expression of *IGF2*-derived intronic pri-miR-483 was reduced before its processing and formation of mature miR-483-3p and miR-483-5p. Based on this result, it is reasonable to consider that miR-493-5p may control pri-miR-483 levels through a regulation mechanism that occurs in the nucleus. Previous works have evidenced that some miRNA fractions are imported back into the nucleus after maturation to regulate gene expression^31,32^. We hypothesized that nuclear miR-493-5p may interact with *IGF2* splicing products from its second intron and pri-miR-483, impeding mature miR-483-3p/5p production.

The oncogenic properties of miR-483-3p have been well described in an interesting study by Veronese et al.^27^. The authors demonstrated that miR-483-3p protected HepG2 cells from apoptosis by targeting BCL2 binding component 3 (*BBC3*), a p53-upregulated modulator of apoptosis. In vivo experiments showed that inhibition of miR-483-3p reduced tumor formation, whereas *IGF2* knockdown did not induce any differences relative to the control. This was consistent with the observations made in the transgenic mouse model overexpressing *Igf2* (without miR-483 expression), which exhibited several abnormalities, such as prenatal overgrowth and disproportional organ development, but did not develop tumors^33^. Finally, Veronese et al. reported overexpression of miR-483-3p in 33% of the HCC samples analyzed in their study. Although a positive correlation between miR-483-3p and *IGF2* expression levels was found in most of the tumors, a significant number of samples exhibited divergent expression of miR-483-3p and *IGF2*, supporting the existence of an *IGF2*-independent mechanism of miR-483-3p expression regulation.

Recent studies have reported the tumor-suppressor activity of miR-493-5p in solid tumors^34–36^. Interestingly, Nakagama’s group showed that miR-493-3p and miR-493-5p were capable of inhibiting settlement of colon cancer cells in the liver parenchyma, in part, by inducing death of the metastasized cells^37^. In line with our current study, Zhao et al.^38^ showed that miR-493-5p downregulation was tightly associated with hepatic tumor progression. In addition, re-expression of miR-493-5p suppressed HCC growth through cell cycle arrest and apoptosis. In another study, Wang et al.^39^ demonstrated that miR-493-5p inhibited cell migration. However, these two studies did not validate the tumor-suppressor activity of miR-493-5p using in vivo models and the potential mechanisms responsible for miR-493-5p silencing were not considered.

In our present work, the effect of miR-493-5p re-expression was relatively variable. Indeed, while miR-493-5p overexpression strongly affected cell growth and invasion in Hep3B cells, this curative effect was more limited in HepG2 and Huh-7 cells (Fig. 4 and Supplementary Fig. 5). The most notable difference between these three cell lines is their tumor protein p53 (*TP53*) status^40^. Hep3B cells are *TP53* deficient, Huh-7 cells exhibit high p53 protein levels due to aberrant stability, and HepG2 cells show normal p53 levels. In addition, the expression levels of miR-493-5p targets (miR-483-3p and *IGF2*), which are higher in Hep3B cells (Fig. 6b, c), could also explain this differential response to miR-493-5p rescue.

A plethora of studies have described the central role played by miRNAs in the development and progression of various diseases, including cancer. Thus far, the mechanisms responsible for altered expression of critical cancer-driver or tumor-suppressor miRNAs have remained relatively poorly described. The present work demonstrated that epigenetic regulation through DNA methylation is essential for controlling miRNA expression. We previously reported epigenetic silencing of another major tumor-suppressor miRNA, miR-148a, in liver cancer cells^41^. Additional works have evidenced the silencing of tumor-suppressor miRNAs through CpG hypermethylation in various types of malignancy^42–44^, reinforcing the thesis that abnormal DNA methylation is critical in miRNA expression deregulation.

The value of epigenetic drugs for reversing aberrant hypermethylation marks and treating solid tumors has been recently considered^45–48^. Our group showed that the use of a demethylating agent was appropriate for reactivation of miR-122 tumor-suppressor and hepatospecific marker genes^49^. In addition, we demonstrated that hypomethylating treatments improved the cytotoxic effect of sorafenib, one of the few molecules approved for therapeutic management of HCC patients. Given that primary liver cancer is associated with a dramatically poor prognosis, primarily because of drug resistance developed by HCC cells to the existing therapies^50^, we evaluated whether miR-493-5p could improve the cytotoxic effect of a panel of anti-cancer drugs in Hep3B cells. Unfortunately, miR-493-5p rescue did not satisfactorily reduce HCC cell growth or viability in response to sorafenib (Supplementary Fig. 6), sunitinib, fluorouracil (5-FU), or cisplatin treatment (data not shown). Although further investigations will be essential to evaluate the benefit of miRNA-based therapies, we are convinced that reactivation of key tumor-suppressor miRNAs may be attractive for development of novel treatments that remain urgently required to tackle tumor progression.

In conclusion, our study demonstrated that miR-493-5p rescue could benefit patients with *IGF2*-miR-483 LOI expression features. Importantly, we highlighted a regulatory mechanism between two imprinted loci, in which the reactivation of epigenetically silenced *MEG3*-derived miR-493-5p could prevent abnormal expression of the *IGF2*-derived oncomir miR-483-3p.

## Materials and methods

### Cell lines, human hepatocytes, and clinical samples

Human HepG2 and Hep3B cells were purchased from the American Type Culture Collection. Human Huh-7 cells were purchased from the RIKEN Bio Resource Center. All cultured cells were maintained in DMEM (Gibco) supplemented with penicillin (50 IU/mL; Gibco), streptomycin (50 µg/mL; Gibco), and 10% fetal bovine serum (FBS; Thermo Scientific). Human cryopreserved hepatocytes were purchased from XenoTech and maintained in a medium composed of William’s Medium E (Gibco), l-glutamine (2 mM), penicillin (50 IU/mL), streptomycin (50 µg/mL), and 10% FBS supplemented with hepatic growth factor (HGF, 25 ng/mL; PeproTech), insulin (5 µg/mL; Sigma) and hydrocortisone 21-hemisuccinate (2 × 10^−7^ M; Sigma). The clinical samples included 18 pairs of primary HCCs and their corresponding non-tumor tissues. None of the patients exhibited hepatitis B (HBV) or C virus (HCV) infection (see Supplementary Table 1 for clinical data).

### Cell transfection and treatment

Hep3B, HepG2, and Huh-7 cells were seeded at a density of 60,000, 80,000, and 70,000 cells/cm^2^ in 35-mm-diameter culture dishes, respectively, and transfected the next day using TransFectin lipid reagent (Bio-Rad Laboratories). The cells were incubated with a transfection mix containing 100 nM miRNA mimic, miRNA inhibitor, or siRNA and 5 µL of TransFectin in a 1.2 mL total volume of serum- and antibiotic-free OptiMEM (Invitrogen) for 5 h. The human miR-493-5p mimics (ID #MC10627, cat. #4464066), miR-483-3p inhibitors (ID #MH12478, cat. #4464084), negative control miRNA mimics (cat. #4464058), and negative control miRNA inhibitors (cat. #4464076) were purchased from Ambion. For stable clone establishment, Hep3B cells were transfected as described above using a miExpress miRNA Expression Clone (cat. #HmiR0211-MR04) and OmicsLink miRNA Expression Clone vectors (cat. #CmiR0001-MR04) from GeneCopoeia to generate miR-493 and control clones, respectively. Transfected cells were selected using 2 µg/mL puromycin dihydrochloride (PubChem CID: 443311) from Sigma (#P9620). Hep3B monoclonal cell lines were generated by limiting dilution and sustained exposure to puromycin. Human IGF2 siRNAs (ID #s7215 and #s7214, siIGF2_A, and siIGF2_B, respectively, cat. #4392420), DNMT1 siRNAs (ID #s4215 and #s4217, siDNMT1_A and siDNMT1_B, respectively, cat. # 4390824), and Silencer Select Negative Control siRNAs (siCtrl_B, cat. #4390843) were purchased from Ambion. AllStars Negative Control siRNAs (siCtrl_A, cat. #1027281) were from Qiagen. The demethylating agent 5-azacytidine (5-AZA; PubChem CID: 9444) was from Sigma (#A2385). The compound was dissolved in phosphate-buffered saline as a 10-mM stock, filtered (0.22 µM), and stored at −20 °C. The cells were treated with 5-AZA at a concentration of 2.5 µM. The drug and medium were replaced daily.

### Total RNA and genomic DNA isolation

mRNA and miRNA were purified using a miRNeasy Mini Kit (Qiagen) according to the manufacturer’s protocol. Total RNA was quantified using a NanoDrop 1000 spectrophotometer (Thermo Scientific), and the integrity of the RNA was evaluated with an Agilent 2100 Bioanalyzer (Agilent Technologies). Genomic DNA was extracted using a GenElute Mammalian Genomic DNA Miniprep Kit (Sigma) and quantified on the NanoDrop 1000 spectrophotometer.

### Gene expression microarray

For assessment of epigenetically silenced miRNAs, total RNA samples were collected from HepG2 cells every 48 h from the beginning of 5-AZA treatment until day 12. For characterization of miR-493-5p targets, total RNAs were collected from Hep3B and HepG2 cells 72 h after cell transfection using miR-493-5p and negative control miRNA mimics. RNA labeling and hybridization were performed using a human microRNA microarray kit (Protocol for Use with Agilent Gene Expression Oligo Microarrays Version 5.7, Agilent Technologies). Microarray slides were scanned in an Agilent Technologies G2505C Microarray Scanner at 3 micron resolution. The raw data were processed using Feature Extraction Software version 10.7.3.1 from Agilent to analyze the array and calculate the intensities of the measured spots. We applied a ≥1.8-fold change in signal intensity to identify significant differences in gene expression after miR-493-5p overexpression. Hierarchical clustering analyses were performed using Pearson correlation. Heat maps were generated using the microarray data analysis tool Multi Experiment Viewer (MeV; http://www.tm4.org/mev.html).

### mRNA and miRNA reverse transcription-quantitative polymerase chain reaction

To evaluate gene expression levels, total RNA was first treated with DNase using a TURBO DNA-free kit (Ambion). Then, cDNAs were synthesized from 1 µg of purified mRNA using SuperScript III Reverse Transcriptase (Invitrogen) according to the manufacturer’s recommendations. SYBR Green RT-qPCR was performed to evaluate the mRNA levels in each sample (Platinum SYBR Green qPCR SuperMix-UDG, Invitrogen) using a Step One Plus Real-time PCR System from Applied Biosystems. After initial denaturation at 95 °C for 2 min, the thermal cycles were repeated 40 times as follows: 95 °C for 15 s and 60 °C for 30 s. The housekeeping genes glyceraldehyde 3-phosphatase dehydrogenase (*GAPDH*) and ribosomal protein S18 (*RPS18*) were used to normalize the cDNA levels. The sequences of the human primers used for gene amplification are shown in Supplementary Table 2. For miRNA analyses, 100 ng of total RNA was reverse-transcribed using a TaqMan miRNA Reverse Transcription Kit from Applied Biosystems (cat. #4366597). The expression levels of mature and primary miRNAs were determined using RT-qPCR with TaqMan Universal PCR Master Mix (Applied Biosystems, cat. #4324018). The PCR conditions were 50 °C for 2 min and 95 °C for 10 min, followed by 40 cycles of 95 °C for 15 s and 60 °C for 1 min. TaqMan probes from Applied Biosystems (cat. #4427975) were used to assess the expression of miR-493-3p (ID #002364), miR-493-5p (ID #001040), miR-483-3p (ID #002339), miR-483-5p (ID #002338), miR-377-3p (ID #000566), and miR-122-5p (ID #002245). The expression levels of mature miRNAs were normalized to the endogenous levels of RNU6B (ID #001093, cat. #4427975). TaqMan probes were used to assess the expression of pri-miR-483 (ID #Hs03293803_pri, cat. #4427012), which was normalized to the endogenous levels of *GAPDH* (ID #Hs99999905_m1, cat. #4331182).

### DNA methylation analysis

Combined bisulfite restriction analysis (COBRA) was used to assess the methylation status of *MEG3*-DMR. An in silico analysis using the UCSC Genome Bioinformatics tool (http://genome.ucsc.edu) was performed to identify CpG sites located upstream of the *MEG3* transcription start site. MethPrimer (http://www.urogene.org/methprimer) was used to design the COBRA primers required to amplify the genomic regions containing the CpGs of interest (Supplementary Table 3). In total, 12 CpG sites were analyzed by COBRA in two distinct regions of *MEG3*-DMR ranging from −1864 to −1593 bp for Region 1 (CpG #1 to #7) and −353 to −58 bp for Region 2 (CpG #8 to #12). Briefly, 1 µg of genomic DNA was subjected to bisulfite modification treatment using an EpiTect Plus kit (QIAGEN). Then, COBRA PCR was performed as follows: after an initial denaturation step at 94 °C for 3 min, the following thermal cycles were repeated 40 times: 94 °C for 10 s, 55 °C for 50 s, and 72 °C for 1 min. Each COBRA PCR was performed in a total volume of 10 µL, which contained 0.5 units of Hot Start Taq polymerase (Takara), 10 pmol of primers, and 1 µL of bisulfite-treated DNA. After PCR amplification, 3 µL of products were digested with three units of restriction enzyme. Last, the restriction products were separated by polyacrylamide gel electrophoresis (PAGE) and visualized by ethidium bromide staining. The bands were densitometrically analyzed using the ImageJ software (v1.50i, National Institutes of Health, USA) to quantify the unmethylated (U) and methylated (M) restriction fragments. The methylation levels were calculated for each locus using the formula (M × 100)/(M + U) and are expressed as a methylation percentage.

### Cell growth assay

For cell growth assessment, HCC cells were seeded at 7500 cells per well in 96-well plates 24 h after transfection. Cell viability was measured at the indicated times using Cell Counting Kit-8 (Dojindo) according to the manufacturer’s instructions (WST-8 assay). The absorbance at 450 nm was measured using a Synergy H4 Microplate Reader system (BioTek). For the evaluation of sorafenib cytotoxicity, miR-493-5p mimic and control mimic-transfected cells were seeded in 96-well plates (10,000 cells/well). The next day, the medium was changed, and the cells were cultured in medium containing different concentrations of sorafenib for 48 h. Cell viability was measured as mentioned above. Sorafenib (Nexavar; PubChem CID: 216239) was purchased from Santa Cruz Biotechnology (#SC-220125).

### Invasion assay

The invasion ability of Hep3B and Huh-7 cells was assessed in 24-well Biocoat Matrigel invasion chambers with an 8-µm pore size (BD Biosciences) according to the manufacturer’s recommended protocol. In brief, 48 h following transfection with miR-493-5p mimics or *IGF2* siRNAs, HCC cells were trypsinized, and 50,000 cells were plated in the upper chamber with serum-free medium. The bottom chamber contained 20% FBS as a chemoattractant. Cells that migrated through the membrane were fixed with methanol 72 h later and stained using a Diff-Quik staining kit (SYSMEX). Invasive cells were automatically counted using a BZ-X700 microscope system (Keyence), and the average number of cells per field was calculated.

### Xenograft establishment and tumor growth assay

Female athymic nude mice were purchased at 4–5 weeks old and housed in isolator units under controlled humidity and temperature, with a 12-h light–dark cycle. The animals were given access to standard sterilized food and water ad libitum. Hep3B cells that stably overexpressed miR-493 or Ctrl sequence were subcutaneously implanted into the right flanks of the mice at a density of 8 × 10^6^ cells via inoculation in DMEM without serum (100 µL/mouse). Tumor nodules were monitored twice a week by palpation using a digital caliper. The tumor size was determined using the formula (length × width^2^)/2 (mm^3^). The animals were euthanized at the study endpoint dictated by the animal care guidelines of our institute, and tumors were immediately collected and snap frozen in liquid nitrogen for storage until RNA and DNA extraction. All animal experiments were performed in accordance with the regulations of the National Cancer Center Institutional Animal Care and Use Committee.

### Immunoblotting

Total protein was extracted using Mammalian Protein Extraction Reagent (M-PER; Thermo Scientific). For each sample, 10 µg of protein was resolved by SDS-PAGE (10%) and transferred to nitrocellulose membranes. The membranes were incubated overnight at 4 °C with anti-IGF2 (Abcam #ab9574), which enabled the detection of IGF2 precursor (approximate molecular weight: 20 kDa), and anti-DNMT1 (Santa Cruz Biotechnology #sc-10219) primary antibodies at a dilution of 1/500. A 1/1500 dilution of anti-β-Tubulin antibody (Sigma #T4026) was used as a loading control. The antigen–antibody complexes were visualized via chemiluminescence using ImmunoStar LD detection reagent from Wako (#290-69904) and scanned with a Fujifilm LAS-3000 imaging system.

### 3′-UTR assay

Dual luciferase reporter plasmids were purchased from GeneCopoeia, where the IGF2 3′-UTR (cat. #CS-MIT234L-MT01) and mutated IGF2 3′-UTR sequences (cat. #CS-MI235L-MT01) were cloned into a pEZX-MT01 vector. Renilla luciferase activity driven by a CMV promoter was used for normalization. Simultaneous cell transfections with 3′-UTR constructs (3 µg) and miR-493-5p mimics (100 mM) were performed in 35-mm-diameter dishes following the experimental procedure described above. Mutated IGF2 vectors and control miRNA mimics were used as negative controls. Cells were collected 24 h after transfection, and protein was extracted using M-PER (Thermo Scientific). The firefly and Renilla luciferase activities were assayed with a Dual-Glo Luciferase Assay System (Promega) using a Synergy H4 Microplate Reader system (BioTek) as recommended by the manufacturer.

### *IGF2* allelic expression analysis

*IGF2* imprinting status was determined based on ApaI digestion of a single-nucleotide polymorphism on exon 9 of human *IGF2* gene (RFLP assay). The primers used for *IGF2* genotyping and imprinting study were as follows: 5′-CTTGGACTTTGAGTCAAATTGG-3′ (forward) and 5′-GGTCGTGCCAATTACATTTCA-3′ (reverse). First, *IGF2* genotype was determined to evaluate whether samples were informative for further analysis of imprinting status. Genomic DNA PCR was performed as follows: after an initial denaturation step at 94 °C for 3 min, the following thermal cycles were repeated 40 times: 94 °C for 15 s, 55 °C for 50 s, and 72 °C for 1 min, followed by a final extension at 72 °C for 5 min. Each reaction was performed in a total volume of 10 µL, which contained 0.5 units of Hot Start Taq polymerase (Takara), 10 pmol of primers, and 100 ng of gDNA. After PCR amplification, 2 µL of products were digested with five units of ApaI restriction enzyme for 15 min at 25 °C. The restriction products were separated by PAGE and visualized by ethidium bromide staining. Samples that were heterozygous for ApaI single nucleotide polymorphism were considered eligible for *IGF2* imprinting analysis. In brief, 100 ng of cDNA was amplified by PCR and digested using ApaI restriction enzyme as described above. Importantly, the absence of genomic DNA contamination in DNase-treated RNA samples was confirmed by an absence of amplification after PCR control reactions that lacked the RT step. Following PAGE, the restriction fragments were densitometrically analyzed using the ImageJ software. *IGF2* LOI (biallelic expression) was defined when the ratio of the more abundant allele to the less abundant allele was <2.

### Statistical analysis

The experimental data are presented as the mean ± SD, except for the in vivo tumorigenicity assay, in which error bars show the SEM. Student’s *t*-test was performed to estimate statistical significance, except for the clinical sample and correlation analysis data. The equality of variances was tested using an F-test, and a correction was performed in the case of unequal variance (Welch *t*-test). All *p*-values were two-tailed. The statistical significance of differences in DNA methylation (HCC cell lines and hepatocytes) and gene expression between the HCC tumors (clinical samples) was assessed using a Mann–Whitney *U* test. Gene expression and DNA methylation correlations were assessed by calculating Spearman’s rank coefficient. The experimental data are representative of at least three independent experiments and were considered statistically significant at *p* < 0.05.

## Accession numbers

Microarray data sets are deposited in the NCBI Gene Expression Omnibus (https://www.ncbi.nlm.nih.gov/geo/) under accession numbers GSE123313 and GSE123314.

## Supplementary information

Supplementary Information.
