# Supplementary material for: MEG3-derived miR-493-5p overcomes the oncogenic feature of IGF2-miR-483 loss of imprinting in hepatic cancer cells
Source: Cell Death Dis. 2019 Jul 18;10(8):553. doi: 10.1038/s41419-019-1788-6 (PMC6639415; doi:10.1038/s41419-019-1788-6)
Supplement: Supplementary file 1 — Supplementary Information. [file 41419_2019_1788_MOESM1_ESM.pdf]

SUPPLEMENTARY INFORMATION

***MEG3*-derived miR-493-5p overcomes the oncogenic feature of *IGF2*-miR-483 loss of imprinting in hepatic cancer cells**

Luc Gailhouste[1], Lee Chuen Liew[1,2], Ken Yasukawa[1,3], Izuho Hatada[4], Yasuhito Tanaka[5], Takashi Kato[3], Hitoshi Nakagama[2,6], Takahiro Ochiya[1]

[1]Division of Molecular and Cellular Medicine, National Cancer Center Research Institute, Tokyo, Japan; [2]Graduate School of Medicine, The University of Tokyo, Tokyo, Japan; [3]Department of Integrative Bioscience and Biomedical Engineering, Graduate School of Advanced Science and Engineering, Waseda University, Tokyo, Japan; [4]Laboratory of Genome Science, Biosignal Genome Resource Center, Institute for Molecular and Cellular Regulation, Gunma University, Maebashi, Japan; [5]Department of Virology and Liver Unit, Nagoya City University Graduate School of Medical Sciences, Nagoya, Japan; [6]National Cancer Center, Tokyo, Japan.

Correspondence: Luc Gailhouste or Takahiro Ochiya.

Present address: Luc Gailhouste, Liver Cancer Prevention Research Unit, RIKEN Center for Integrative Medical Sciences, 2-1 Hirosawa, Wako, Saitama 351-0198, Japan. Email: luc.gailhouste@riken.jp; Takahiro Ochiya, Department of Molecular and Cellular Medicine, Institute of Medical Science, Tokyo Medical University, 6-7-1 Shinjuku-ku, Tokyo 160-0023, Japan. Email: tochiya@tokyo-med.ac.jp.

Supplementary Table 1. Clinical and pathological features of the 18 HCC cases

Supplementary Table 2. List of the primers used for real-time quantitative PCR

Supplementary Table 3. Human primers used for COBRA

Supplementary Figure 1. Validation of miR-493-5p re-expression and miR-483-3p knockdown in hepatic cell lines

Supplementary Figure 2. *MEG3*-DMR methylation profiles in human hepatocytes and expression levels of *MEG3*-miR-493-3p/5p in Huh-7 cells after epigenetic unmasking

Supplementary Figure 3. Effect of *DNMT1* knockdown on *MEG3*-miR-493-5p expression and methylation levels in HCC cells

Supplementary Figure 4. Measurement of *MEG3*-miR-493-3p and *IGF2*-miR-483-3p expression levels in the clinical samples from HCC patients

Supplementary Figure 5. Invasive abilities of miR-493-5p-rescued Huh-7 cells

Supplementary Figure 6. HCC cell response to sorafenib treatment after miR-493-5p rescue

Supplementary Figure 7. miR-493 Hep3B clone characterization and *in vivo* tumor growth assay

Supplementary Figure 8. Gene enrichment analysis in miR-493-5p-rescued HCC cells

Supplementary Figure 9. Expression levels of *IGF2*, miR-483-3p, and miR-483-5p in response to 5-AZA demethylating treatment

Supplementary Figure 10. miR-493-5p and miR-483-3p expression levels from a public database

Supplementary Figure 11. Effect of *IGF2* experimental knockdown on HCC cell growth

Supplementary Table 1. Clinical and pathological features of the 18 HCC cases

	No. of cases
Gender	
Male	12 (66.7 %)
Female	6 (33.3 %)
Age	
Median (range)	74 (51-87)
< 70	7 (38.9 %)
> 70	11 (61.1 %)
Serology	
HBV	0
HCV	0
Alcohol consumption	
No	12 (66.7 %)
Moderate	4 (22.2 %)
Yes	8 (11.1 %)
Tumor size	
< 5 cm	9 (50 %)
Median (range)	6.7 cm (5.0-15.5)
> 5 cm	9 (50 %)
Median (range)	3.2 cm (2.6-4.8)
Tumor differentiation	
Moderately	12 (66.7 %)
Poorly	6 (33.3 %)
Plasma α-fetoprotein	
< 300 ng/mL	13 (72.2 %)
> 300 ng/mL	5 (27.8 %)

Supplementary Table 2. List of the primers used for real-time quantitative PCR

Gene	Name	Sense	Antisense
MEG3	Maternally expressed 3	aaggaccacctcctctccat	gtcagttccggtcctcttca
IGF2	Insulin like growth factor 2	gcatcgttgaggagtgctgt	acggggtatctggggaagtt
DNMT1	DNA methyltransferase 1	atgcttacaaccgggaagtg	tgaacgcttagcctctccat
GAPDH	Glyceraldehyde 3-phosphatase dehydrogenase	gagtcaacggatttggtcgt	ttgattttggagggatctcg
RPS18	Ribosomal protein S18	gaggatgaggtggaacgtgt	ggacctggctgtattttcca

The primers were designed using Primer3 v.0.4.0 (http://bioinfo.ut.ee/primer3-0.4.0/primer3) and purchased from Invitrogen.

Supplementary Table 3. Human primers used for COBRA

Target name	Sense	Antisense	CpG #
MEG3 #01	TATGTTTTTGTGGGGT TGTA	ACCACAATATTAATA ACTAAAAAACAA	1-3
MEG3 #02	TTTTAGTTATTAATAT TGTGGTTAATAAA	ACAACCTATAAAACT TACCAAAAAC	4-7
MEG3 #03	TTTTTTGAATAATAAG AGAAAGTATGATTT	CACCCAAATTACAAC AAAAAAAA	8-10
MEG3 #04	TTTTTTTGTTGTAATTT GGGTG	AAAAACTAACTAAAT AAAATTTATATAAAA	11-12

All primers were designed using the UCSC Genome Bioinformatics Site (http://genome.ucsc.edu) and MethPrimer (http://www.urogene.org/methprimer), and were purchased from Invitrogen.

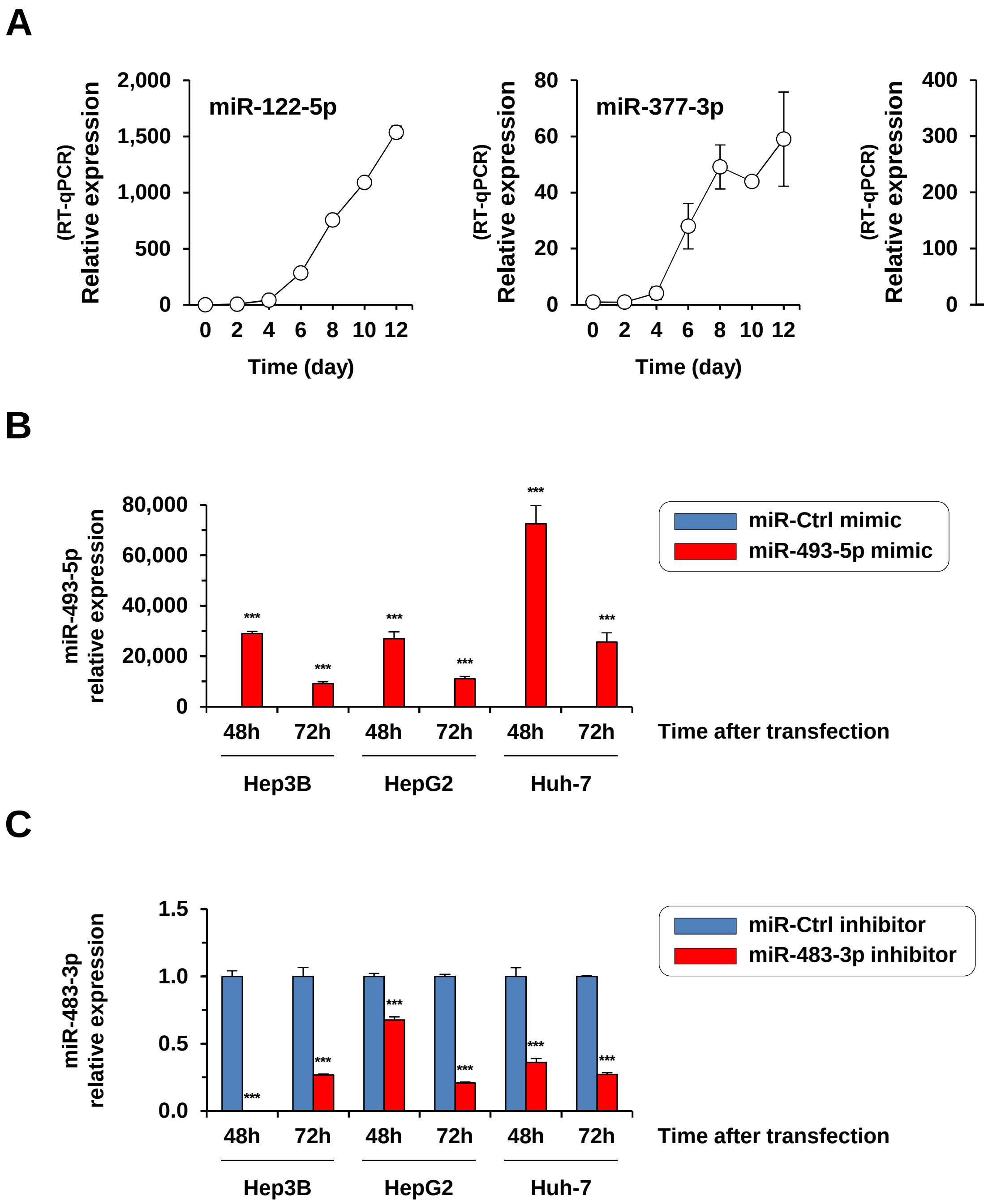

Supplementary Figure 1

Supplementary Figure 1. Validation of miR-493-5p re-expression and miR-483-3p knockdown in hepatic cell lines

(**A**) Confirmation of miR-493-5p re-expression in the samples used for microarray analysis. The expression profiles of 3 representative miRNAs extracted from the top 15 after epigenetic unmasking (miR-122-5p, miR-377-3p, and miR-493-5p) were determined via RT-qPCR in treated HepG2 cells (2.5 µM 5-AZA) at the indicated times. The time course data shown in this figure are representative of 3 independent experiments. Non-treated cells were used as control (D0). (**B**) Confirmation of miR-493-5p experimental rescue. Mature miR-493-5p expression levels were measured via RT-qPCR after miR-493-5p overexpression using miRNA mimics in Hep3B, HepG2, and Huh-7 cells. (**C**) Confirmation of miR-483-3p experimental knockdown. Mature miR-483-3p expression levels were measured via RT-qPCR after miR-483-3p inhibition using miRNA inhibitors in Hep3B, HepG2, and Huh-7 cells.

The data shown are the mean ± SD. Significant differences in miRNA expression were reached at ***($p < 0.001$) (t-test).

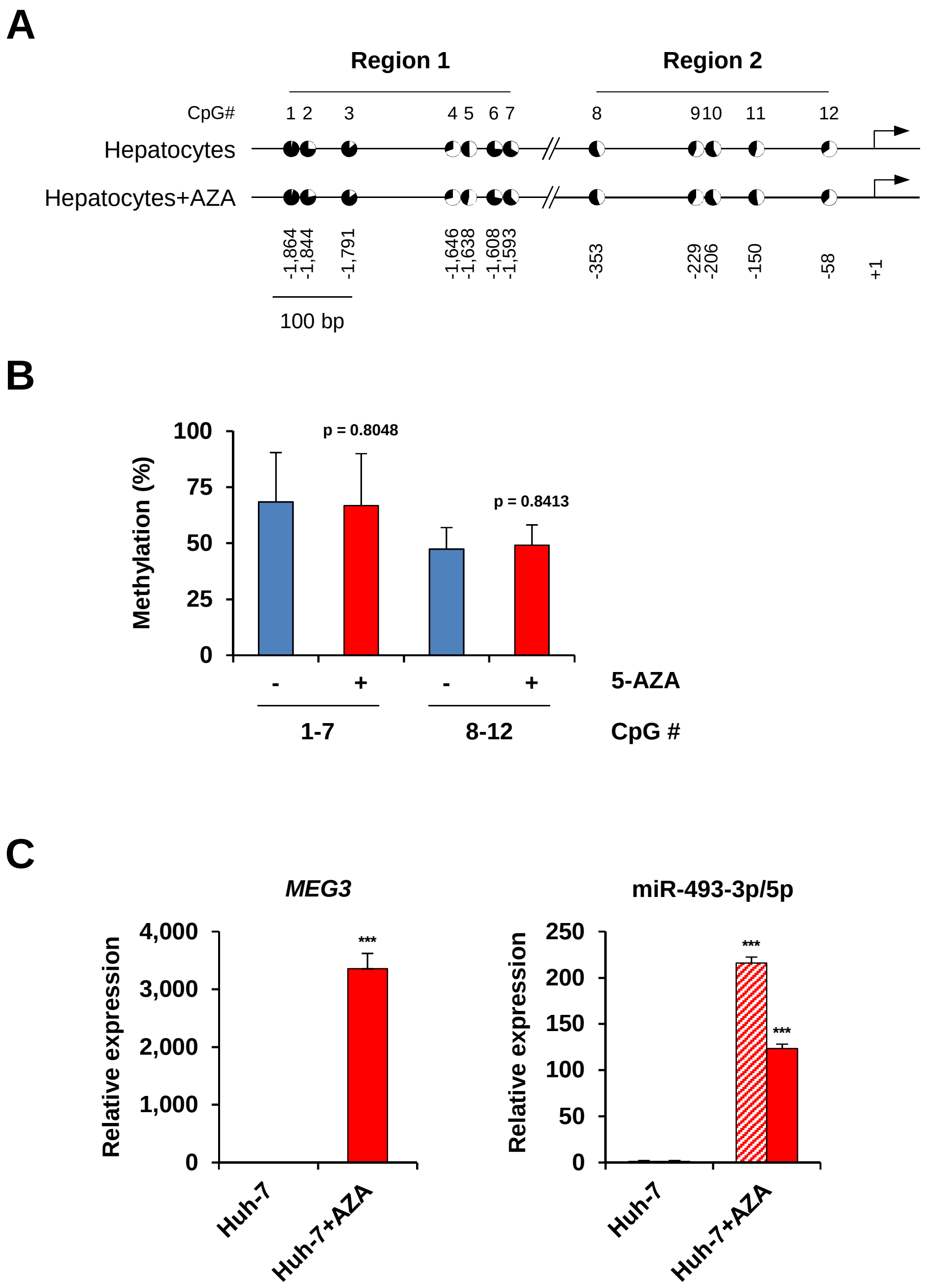

Supplementary Figure 2

Supplementary Figure 2. *MEG3*-DMR methylation profiles in human hepatocytes and expression levels of *MEG3*-miR-493-3p/5p in Huh-7 cells after epigenetic unmasking

(**A**) Comparison of the methylation levels of *MEG3*-DMR in human hepatocytes before and after epigenetic unmasking, evaluated using COBRA. The circles represent in black the methylation percentages calculated for each CpG site. (**B**) Quantification of *MEG3*-DMR methylation status after 5-AZA treatment. The methylation ratios (%) represent the average values calculated for CpG #1 to #7 and CpG #8 to #12, which delimited Region 1 and Region 2, respectively. Genomic DNA was extracted after demethylating treatment with 2.5 µM 5-AZA for 10 days. (**C**) Expression levels of *MEG3*, miR-493-3p, and miR-493-5p after epigenetic unmasking in Huh-7 cells. RNAs were extracted after 10 days of treatment (5-AZA). Non-treated cells were used as controls.

Histograms represent the mean ± SD. Significant differences relative to the control cells were reach at ***($p < 0.001$) (t-test).

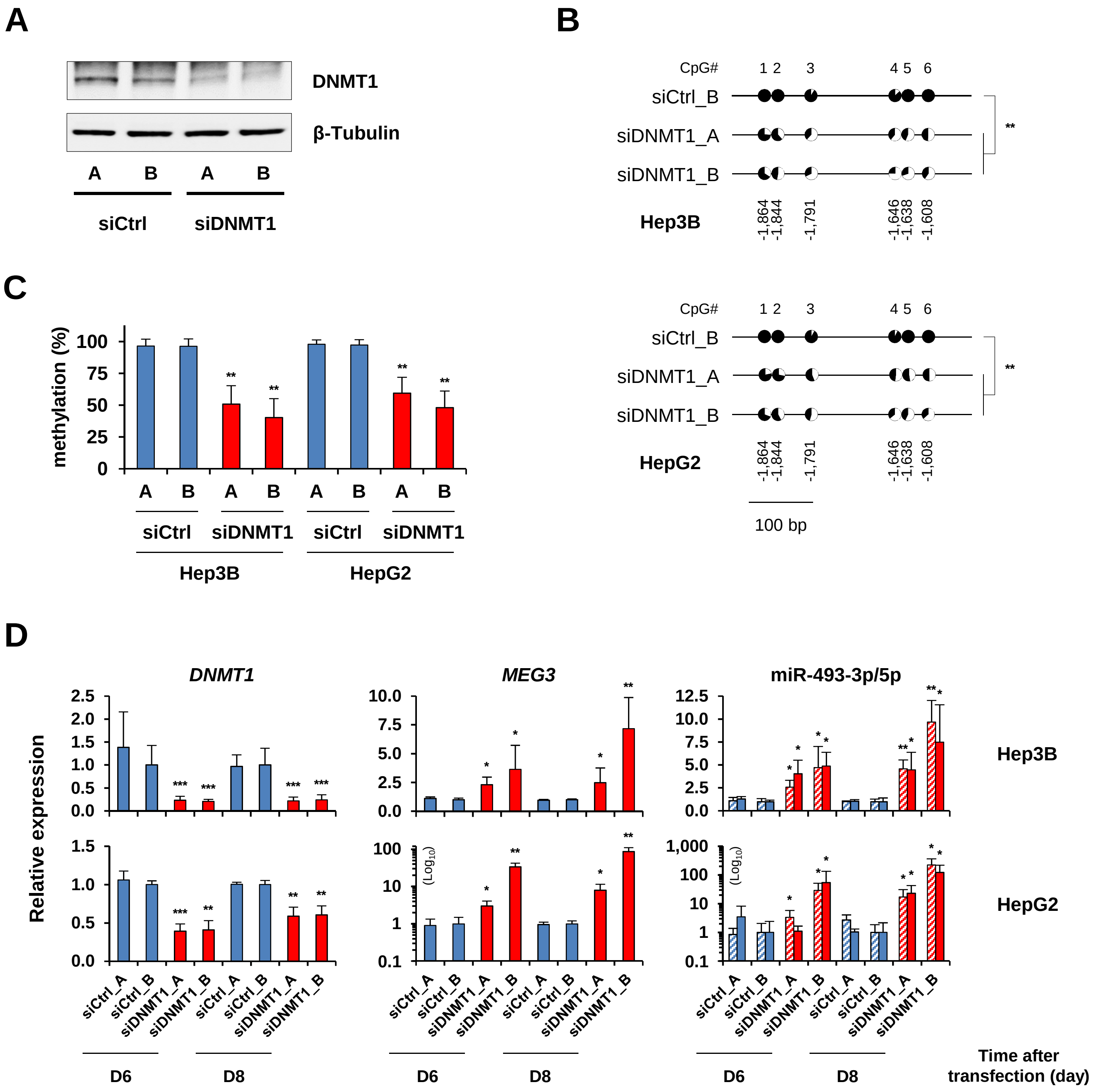

Supplementary Figure 3

Supplementary Figure 3. Effect of *DNMT1* knockdown on *MEG3*-miR-493-5p expression and methylation levels in HCC cells

(A) DNMT1 protein levels in *DNMT1*-knockdown HepG2 cells. Proteins were extracted 8 days after transfection. β-Tubulin was used as the loading control for immunoblots. Two distinct siRNAs were used to target *DNMT1* (siDNMT1_A and siDNMT1_B), and two scrambled siRNAs were used as negative controls (siCtrl_A and siCtrl_B). (**B**) Methylation status of the *MEG3*-DMR in HepG2 and Hep3B cells, evaluated using COBRA after experimental silencing of *DNMT1*. (**C**) Quantification of methylated DNA (%) after knockdown of *DNMT1* in HCC cells. (**E**) Relative expression of *DNMT1*, *MEG3*, miR-493-3p, and miR-493-5p following *DNMT1* silencing in HepG2 and Hep3B cells. Gene expression levels were measured 6 and 8 days after transfection.

The histograms shown in the figure represent the mean ± SD. Significant differences versus control transfected cells (siCtrl): *($p < 0.05$); **($p < 0.01$); ***($p < 0.001$) (t-test).

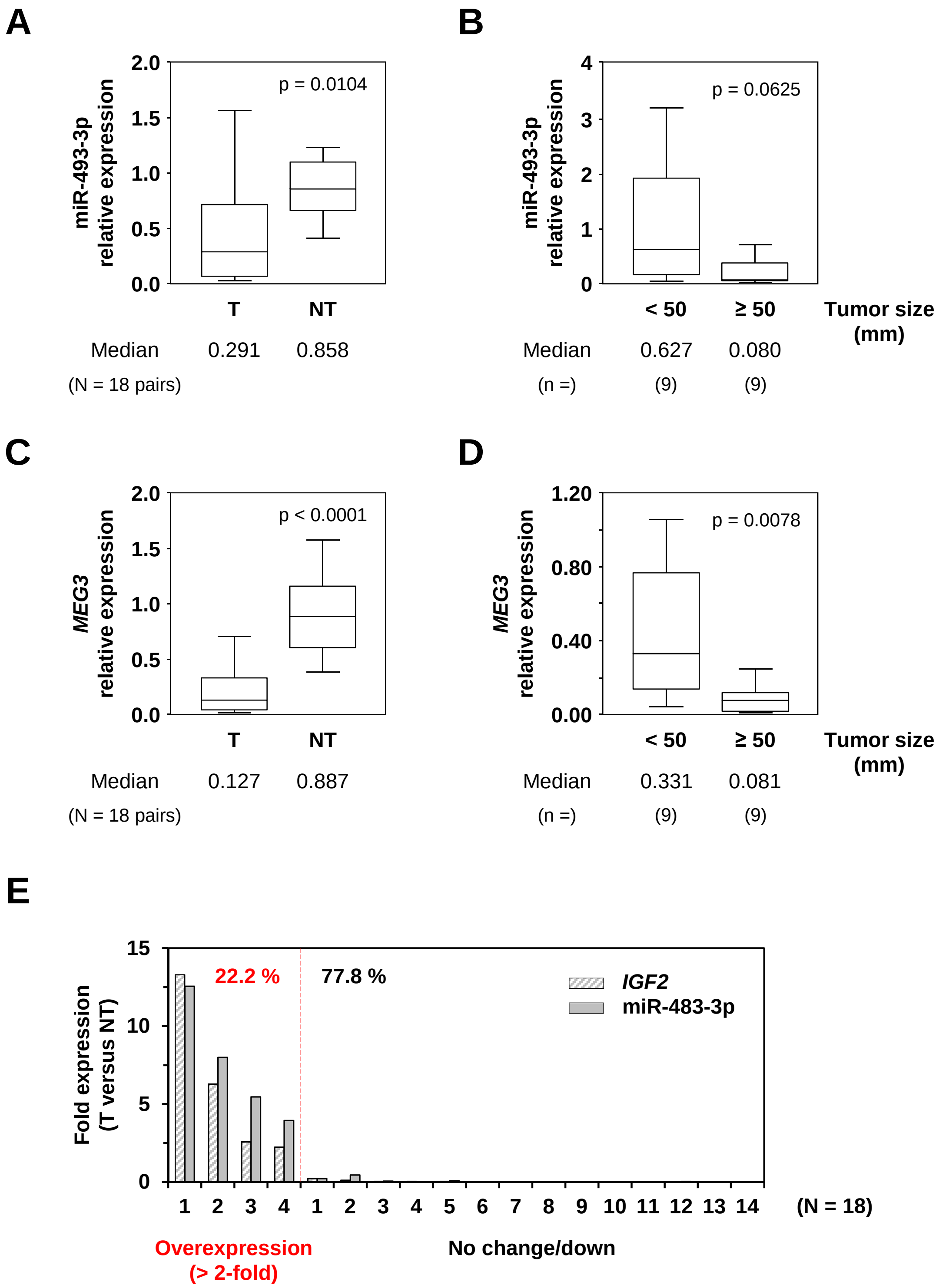

Supplementary Figure 4

Supplementary Figure 4. Measurement of *MEG3*-miR-493-3p and *IGF2*-miR-483-3p expression levels in the clinical samples from HCC patients

(**A**)-(**C**) Expression levels of miR-493-3p (**A**) and *MEG3* (**C**) in clinical samples. Boxplots illustrate the differential expression between 18 primary HCC samples (T) and their corresponding paired non-tumor tissues (NT). (**B**)-(**D**) Comparison of miR-493-3p (**B**) and *MEG3* expression (**D**) between moderate and advanced liver cancer. Advanced HCC tumors were defined as tumors with a size superior or equal to 5 cm. (**E**) Correlated overexpression of miR-483-3p and *IGF2* in HCC tumor samples versus surrounding non-neoplastic tissues (T/NT). 4 of the 18 HCC tumors showed concomitant overexpression (> 2-fold) of miR-483-3p and *IGF2*.

The Mann-Whitney U test was used to calculate the p-value displayed with the boxplots.

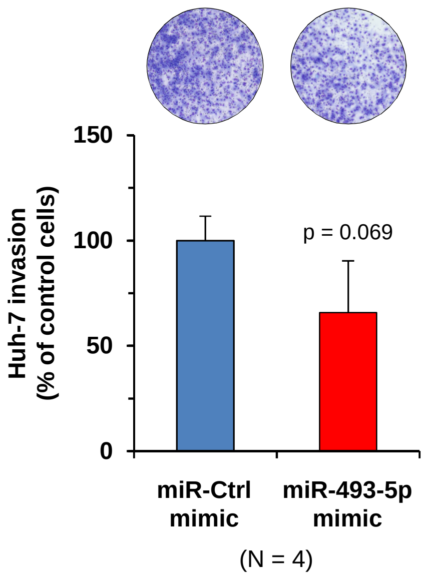

Supplementary Figure 5

Supplementary Figure 5. Invasive abilities of miR-493-5p-rescued Huh-7 cells

Huh-7 cells were transfected with miR-493-5p mimics and control mimics (miR-Ctrl mimic). Transfected cells were plated into invasion chambers coated with Matrigel (upper chamber), and 20 % FBS was used as a chemoattractant (bottom chamber). Cells that migrated through the Matrigel-coated membrane were counted after 72 hours. The histograms show the mean ± SD (N = 4 distinct assays). A *t*-test was performed to calculate the p value.

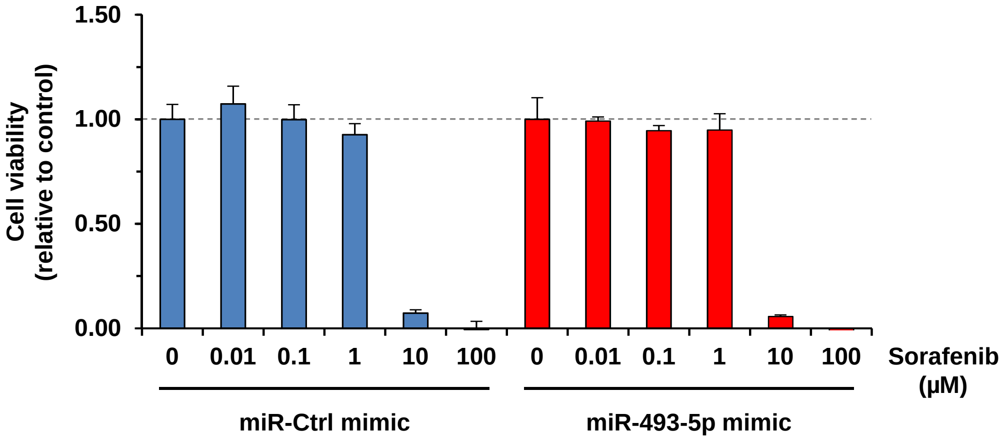

Supplementary Figure 6

Supplementary Figure 6. HCC cell response to sorafenib treatment after miR-493-5p rescue

Hep3B cells were transfected with miR-493-5p mimics and control mimics (miR-Ctrl mimic). Cell viability was measured after sorafenib treatment (48 hours) at the indicated concentrations. Histograms show the mean ± SD. No modification in sorafenib response was observed in miR-493-5p-rescued cells.

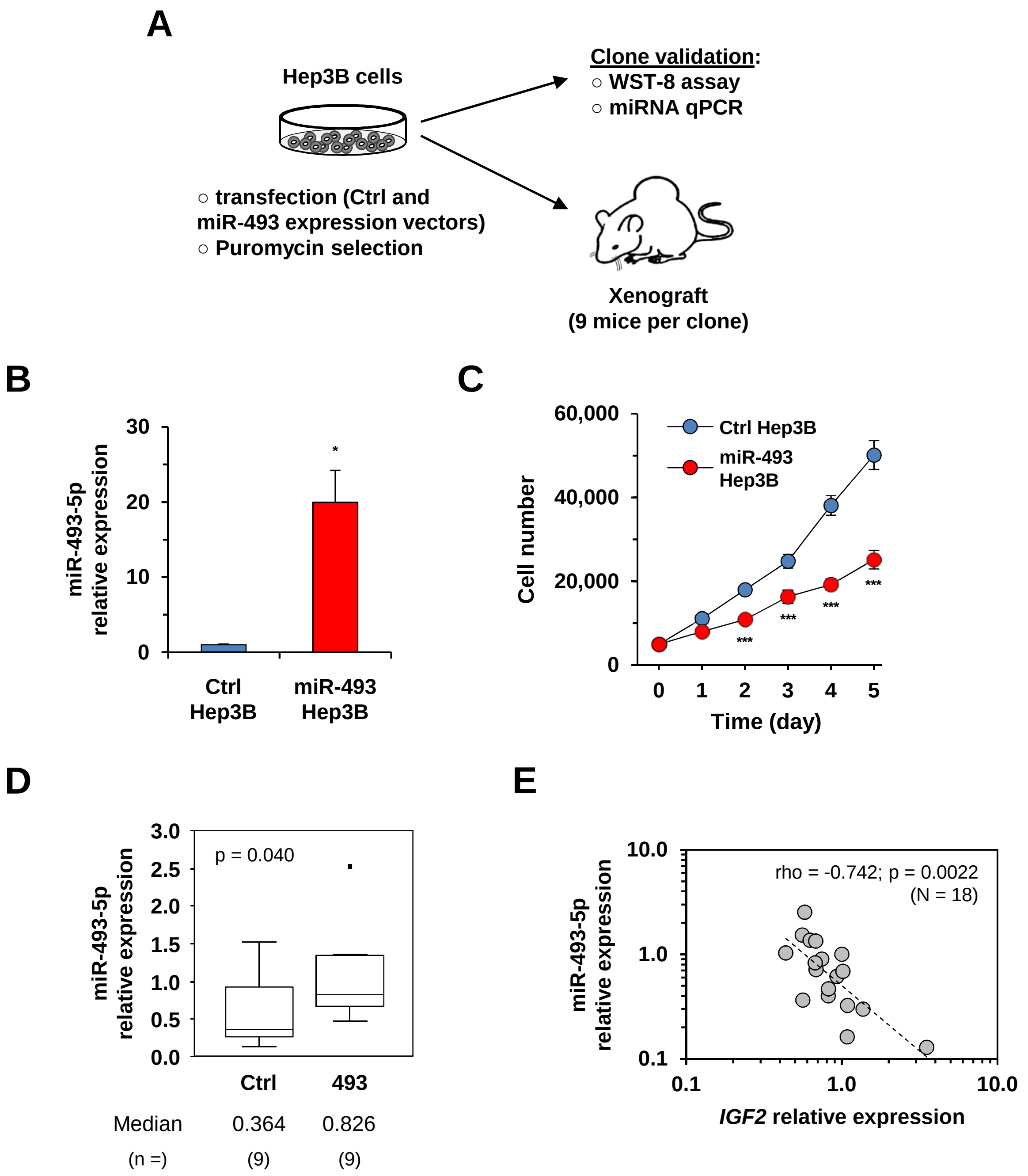

Supplementary Figure 7

Supplementary Figure 7. miR-493 Hep3B clone characterization and *in vivo* tumor growth assay

(**A**) Schematic outline of the assessment of miR-493-5p tumor-suppressor activity *in vivo*. Hep3B cells with stable re-expression of miR-493-5p were first generated and validated *in vitro*. (**B**) miR-493-5p expression level measured in Hep3B cells after puromycin selection (2 µg/mL) and cloning. RT-qPCR data showed stable overexpression of miR-493-5p in cells transfected with the miRNA Expression Clone vector (miR-493 Hep3B) compared with cells that received the control vector (Ctrl Hep3B). The histograms represent the mean ± SD. Significance: *($p < 0.05$) (t-test). (**C**) Hep3B cell growth after stable re-expression of miR-493-5p. The number of cells was estimated at the indicated times using cell viability assays. The data represent the mean ± SD. Significance: ***($p < 0.001$) (t-test). (**D**) Expression levels of miR-493-5p in Hep3B tumors. Tumors were resected at the end of the experiment for RT-qPCR analysis. Boxplots illustrate the differential expression of miR-493-5p between the control group mice and the mice that received cells re-expressing miR-493-5p. Significance (Mann-Whitney U test): *$p < 0.05$. (**E**) Scatter plots of Spearman's correlation coefficient analysis comparing miR-493-5p and *IGF2* expression levels, measured by RT-qPCR in Hep3B tumors.

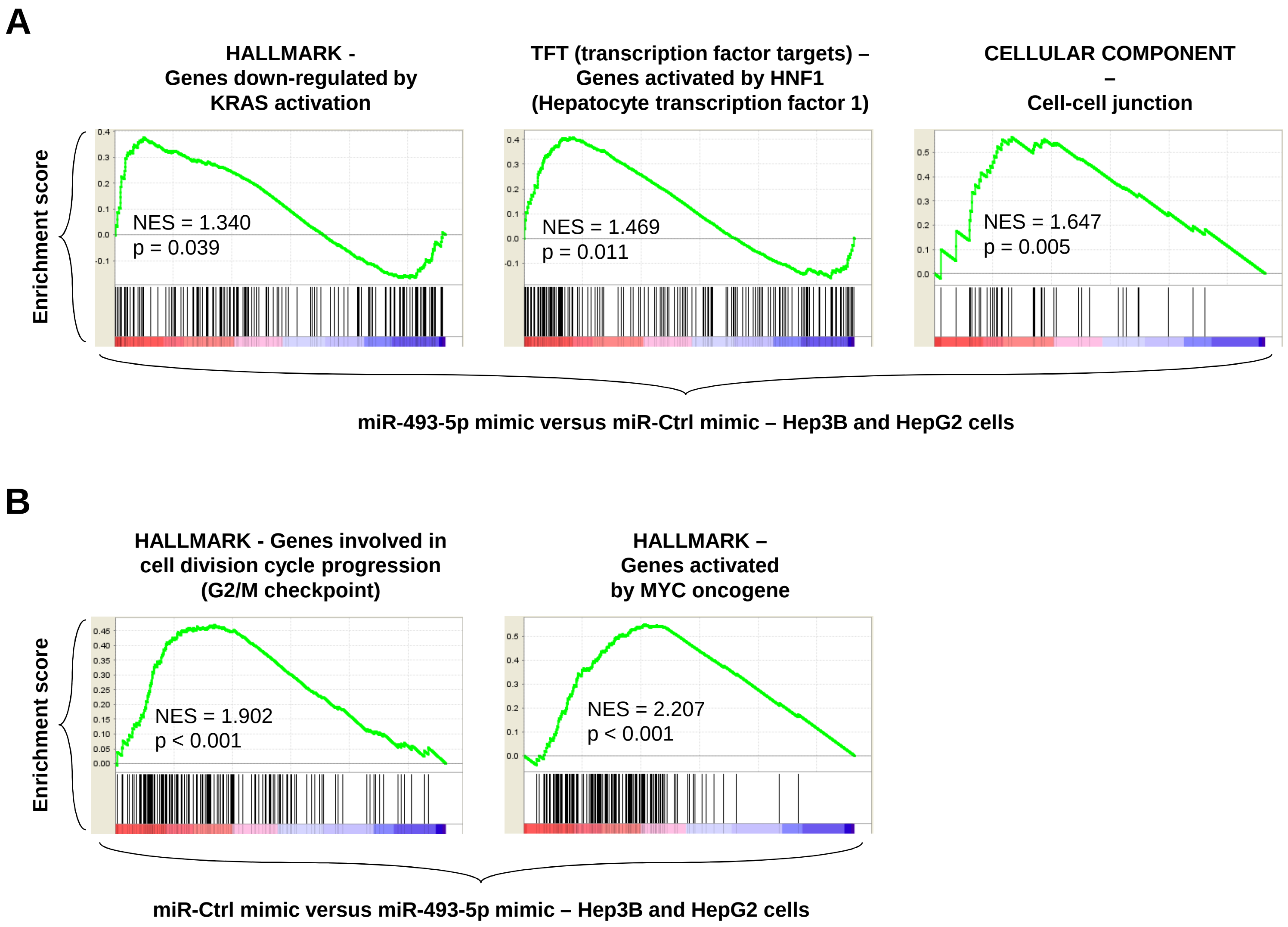

Supplementary Figure 8

Supplementary Figure 8. Gene enrichment analysis in miR-493-5p-rescued HCC cells

Gene Set Enrichment Analysis (GSEA) was performed to assess the effect of miR-493-5p rescue in Hep3B and HepG2 cells (http://software.broadinstitute.org/gsea/index.jsp). Gene expression data from GSE123313 were used for the analysis. (**A**) Enrichment plots for KRAS-downregulated genes, hepatocyte transcription factor 1 (HNF1)-activated genes, and cell-cell junction-related genes in cells overexpressing miR-493-5p versus control cells (miR-Ctrl-mimic). (**B**) Enrichment plots for cell cycle progression genes and MYC-activated genes in control cells versus cells overexpressing miR-493-5p. NES, normalized enrichment score.

A

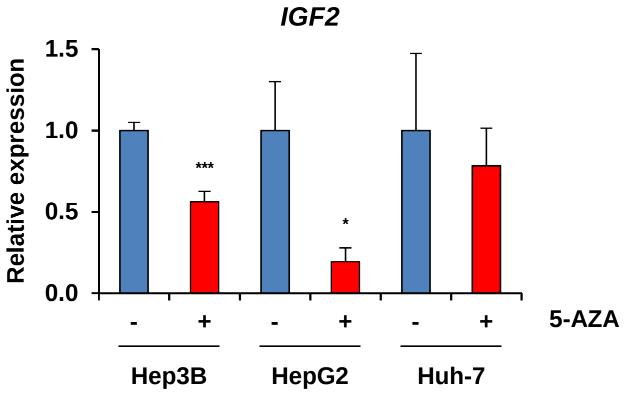

B

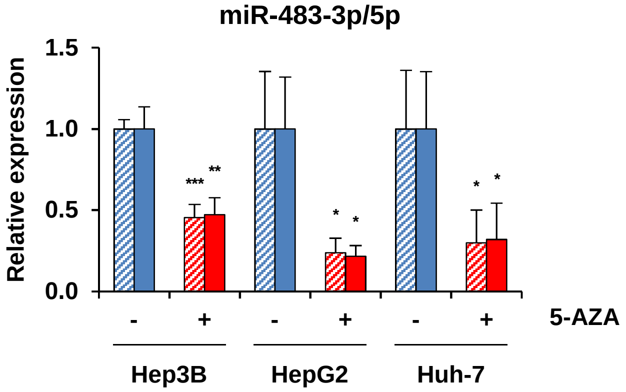

Supplementary Figure 9

Supplementary Figure 9. Expression levels of *IGF2*, miR-483-3p, and miR-483-5p in response to 5-AZA demethylating treatment

(**A-B**) Expression levels of (**A**) *IGF2* and (**B**) miR-483-3p/5p after 5-AZA exposure. The relative mRNA and mature miRNA expression levels were determined by RT-qPCR. Total RNA was extracted 10 days after 2.5 µM 5-AZA treatment. Non-treated HCC cells were used as controls. Histograms represent the mean ± SD. Significant differences relative to the control cells were reached at *($p < 0.05$), **($p < 0.01$), and ***($p < 0.001$) (t-test).

A

B

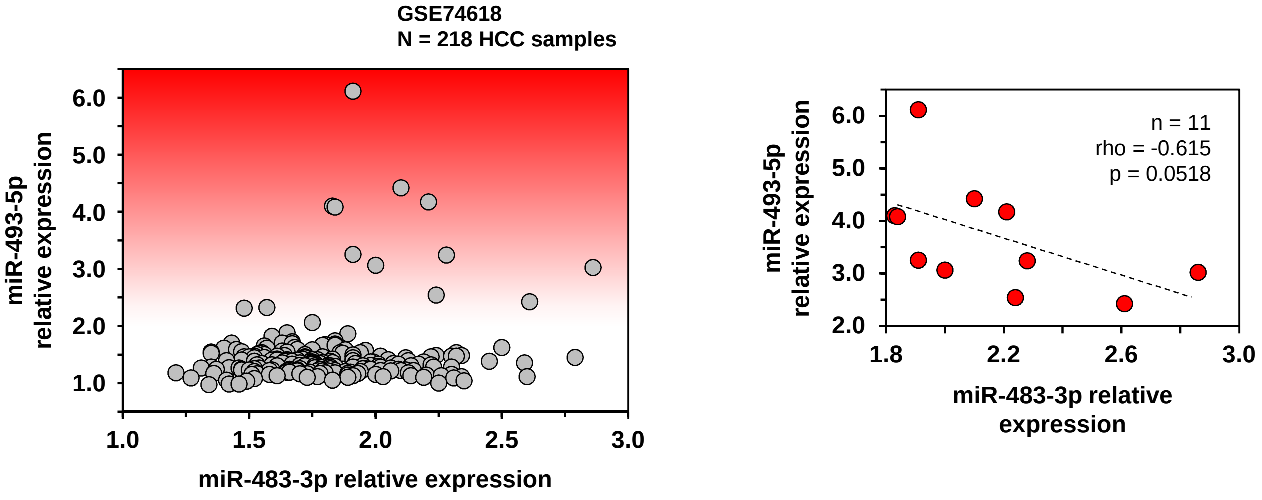

Supplementary Figure 10

Supplementary Figure 10. miR-493-5p and miR-483-3p expression levels from a public database

(**A**) Expression levels of miR-493-3p and miR-483-3p in 218 human HCC tumors samples. GSE74618 dataset from Gene Expression Omnibus (GEO) was used for the analysis. The expression level of miR-493-5p was low and homogenous (median value, 1.33). (**B**) Scatter plots of Spearman's correlation coefficient analysis between miR-493-5p and miR-483-3p expression levels in 11 tumor samples exhibiting significant miR-493-5p expression (median value, 3.25).

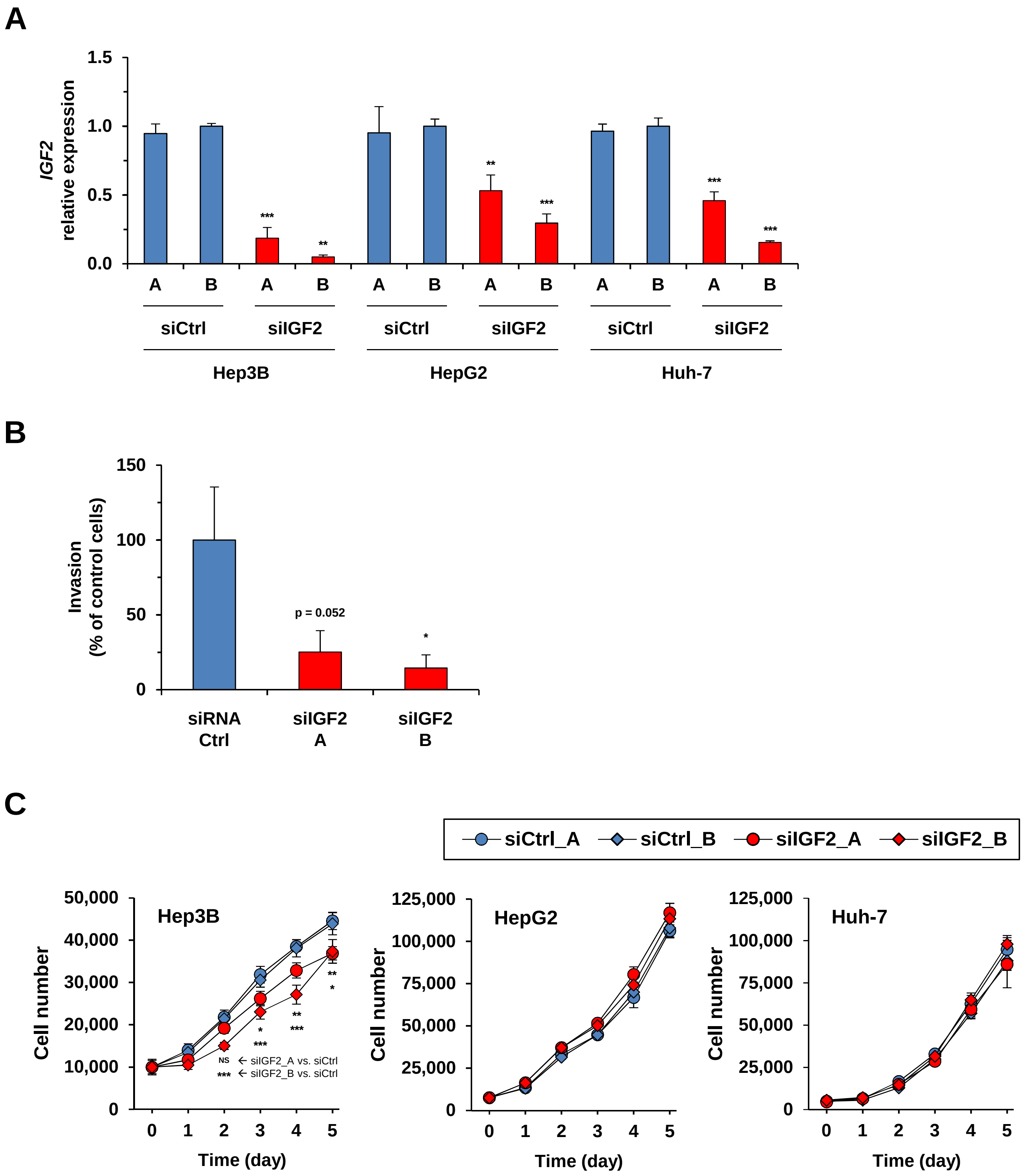

Supplementary Figure 11

Supplementary Figure 11. Effect of *IGF2* experimental knockdown on HCC cell growth

(**A**) *IGF2* expression levels in liver cancer cell lines, determined by RT-qPCR. Total RNA was extracted from the cells 72 h after transfection using 2 distinct siRNAs targeting IGF2 (siIGF2_A and siIGF2_B) and two control siRNAs (siCtrl_A and siCtrl_B). (**B**) Invasive abilities of Hep3B cells after *IGF2* knockdown. Briefly, 48 hours after transfection, Hep3B cells were plated into invasion chambers coated with Matrigel (upper chamber), and 20 % FBS was used as a chemoattractant (bottom chamber). Cells that migrated through the membrane were counted after 72 hours. (**C**) Tumor cell growth evaluated after *IGF2* silencing. The number of cells was estimated at the indicated times using a cell viability assay.

The data shown in this figure represent the mean ± SD. Significant differences in HCC cell migration, cell growth, and gene expression were evaluated with a t-test: *($p < 0.05$), **($p < 0.01$), and ***($p < 0.001$). NS, not significant.
